# Effect of a =X-NH-Fragment, (X = C, N), on *Z*/*E* Isomerization and ON/OFF Functionality of Isatin Arylhydrazones, ((Arylamino)Methylene)Indolin-2-Ones and Their Anions

**DOI:** 10.3390/molecules25133082

**Published:** 2020-07-06

**Authors:** Pavol Tisovský, Klaudia Csicsai, Jana Donovalová, Róbert Šandrik, Róbert Sokolík, Anton Gáplovský

**Affiliations:** Faculty of Natural Sciences, Institute of Chemistry, Comenius University, Ilkovičova 6, Mlynská dolina CH-2, SK-842 15 Bratislava, Slovakia; klaudia.jakusova@uniba.sk (K.C.); jana.donovalova@uniba.sk (J.D.); sandrik2@uniba.sk (R.Š.); robert.sokolik@uniba.sk (R.S.); anton.gaplovsky@rec.uniba.sk (A.G.)

**Keywords:** ON/OFF switches, photoisomerization, hydrazo-azo tautomerization, kinetics, anions of isatin arylhydrazones, ((arylamino)methylene)indolin-2-ones

## Abstract

The subject of this work was the study of thermally and photochemically stimulated *Z* ↔ *E* isomerization and hydrazo ↔ azo tautomerism of *Z*- and *E*-isomers of isatin arylhydrazones and ((arylamino)methylene)indolin-2-ones and their anions. Using NMR, UV-Vis spectroscopy, kinetic measurements, and HPLC, we studied the relationship of structure, (*Z*- and *E*-isomers), of these compounds and hydrazo=azo tautomerism. The ON/OFF functionality of these compounds and their anions using light to stimulate switching between ON and OFF states was investigated. We pointed out the characterization of the effect of =N- and =CH- structural fragments and aryl structure on ON and OFF states of isatin arylhydrazones and ((arylamino)methylene)indolin-2-ones.

## 1. Introduction

Photochromism is defined as a reversible color change induced in a compound by absorption of electromagnetic radiation [1]. Compounds falling under the P-type derivatives category have attracted attention because of their huge application potential in the field of optics and electronics [2,3,4,5]. Although most widely used photochromic derivative classes use UV light for phototransformation, only hemithioindigos fall within the pure Vis-Vis photoswitch class. This hinders the application potential in biomedical and material science because UV light can damage healthy cells and degrade many macromolecular systems [6]. Therefore, the development of new easily synthesized Vis-Vis (Vis-NIR) T- and P-type photochromic dyes still challenges current photochemistry. In recent years hydrazones attract more and more attention due to their unique properties such as facile synthesis, high stability, and application in various fields (photo- and thermo-sensitive supramolecular arrangements and as colorimetric or fluorescent chemosensors) [7,8,9,10]. These compounds belong to photochromic compounds that they use most often light-induced isomerization around double bonds. The properties of these molecules are a prerequisite for their practical application as molecular switches. Hydrazones represent the simplest switch that can distinguish between two states: ON/OFF.

Herein, we report on a systematic study of the behavior and solution switching of the class of isatin arylhydrazones and ((arylamino)methylene)indolin-2-ones. The modifications on phenyl groups of hydrazone part were conducted with the goal to affects the acid-base properties of the NH hydrogens of these compounds and their switching properties.

## 2. Results and Discussions

### 2.1. Synthesis of Studied Compounds

**Scheme 1:**
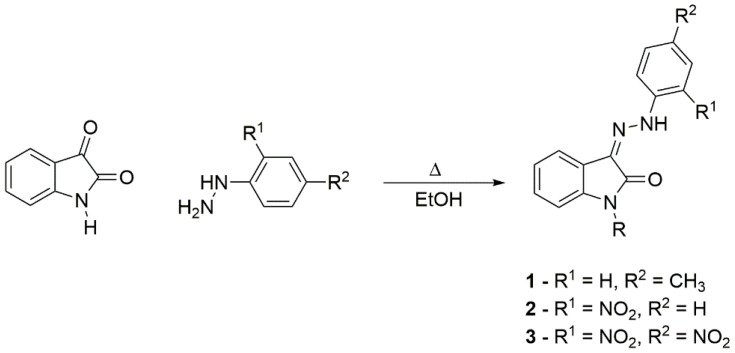
Synthesis of hydrazones **1**–**3**.

**Scheme 2:**
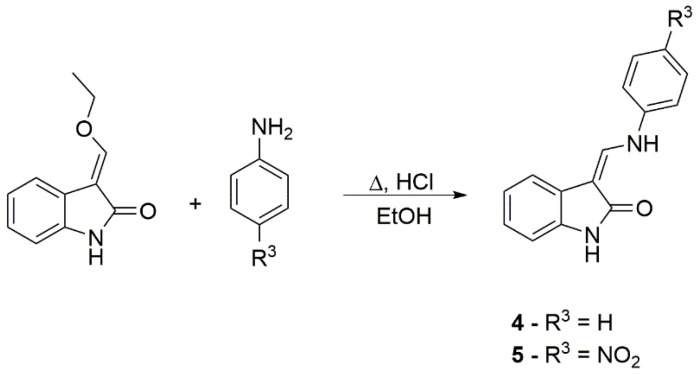
Synthesis of ((arylamino)methylene)indolin-2-ones **4** and **5**.

Compounds **1**–**3** were prepared by a condensation reaction of isatin with the corresponding hydrazine (Scheme 1). The corresponding products were isolated in high yields (68–85%).

((Arylamino)methylene)indolin-2-ones **4** and **5** were prepared by reaction of (ethoxy-methylene)indolin-2-one with the corresponding aniline under acidic conditions (Scheme 2). The corresponding products were isolated in 72 and 75% yields, respectively.

### 2.2. UV-Vis Spectra

#### 2.2.1. Isatin Aryl Hydrazones

**Figure 1:**
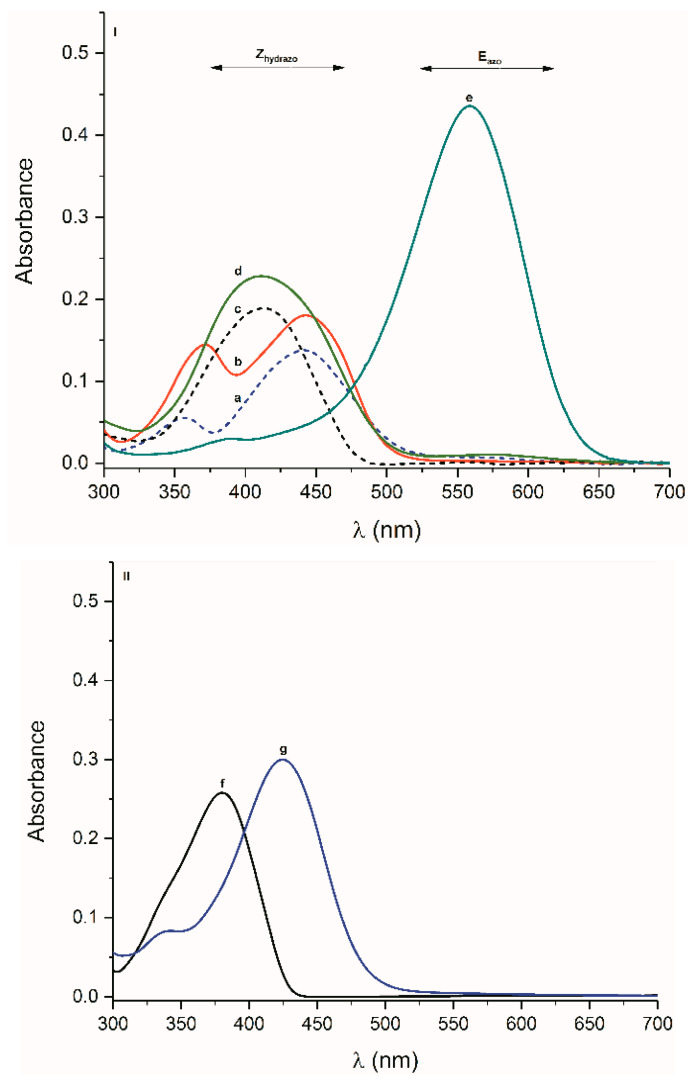
UV-Vis spectra of *Z*- and *E*-isomers (1 × 10^−5^ mol·dm^−^^3^) of compounds: (**I**) (a) **2*E***, (b) **2Z**, (c) **1**, (d) **3Z**, (e) **3*E***; (**II**) (f) **4**, (g) **5** in DMF.

The characteristic ππ* and charge transfer (CT) absorptions of the *E-* and *Z*-isomers of isatin arylhydrazones were in the wavelength range from 350 to 600 nm. The positions of the absorption maxima depended on the electron structure of the hydrazone aryl group and in the case of the *E*-isomers, also on the influence of the aryl moiety on the planarity of the hydrazone *E*-isomer (Figure 1).

**Figure 2:**
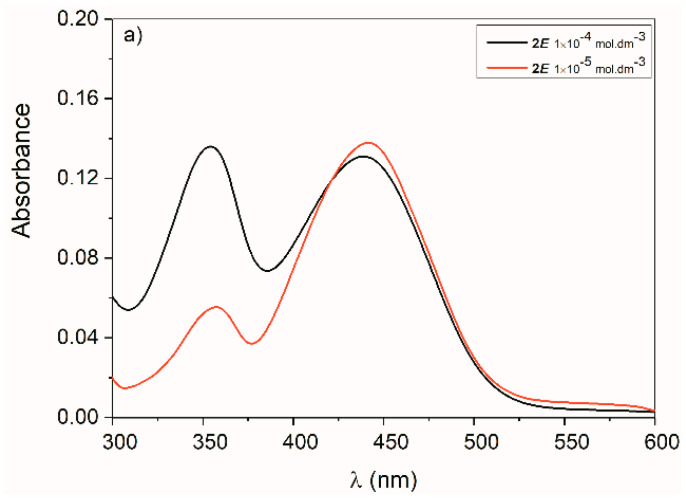

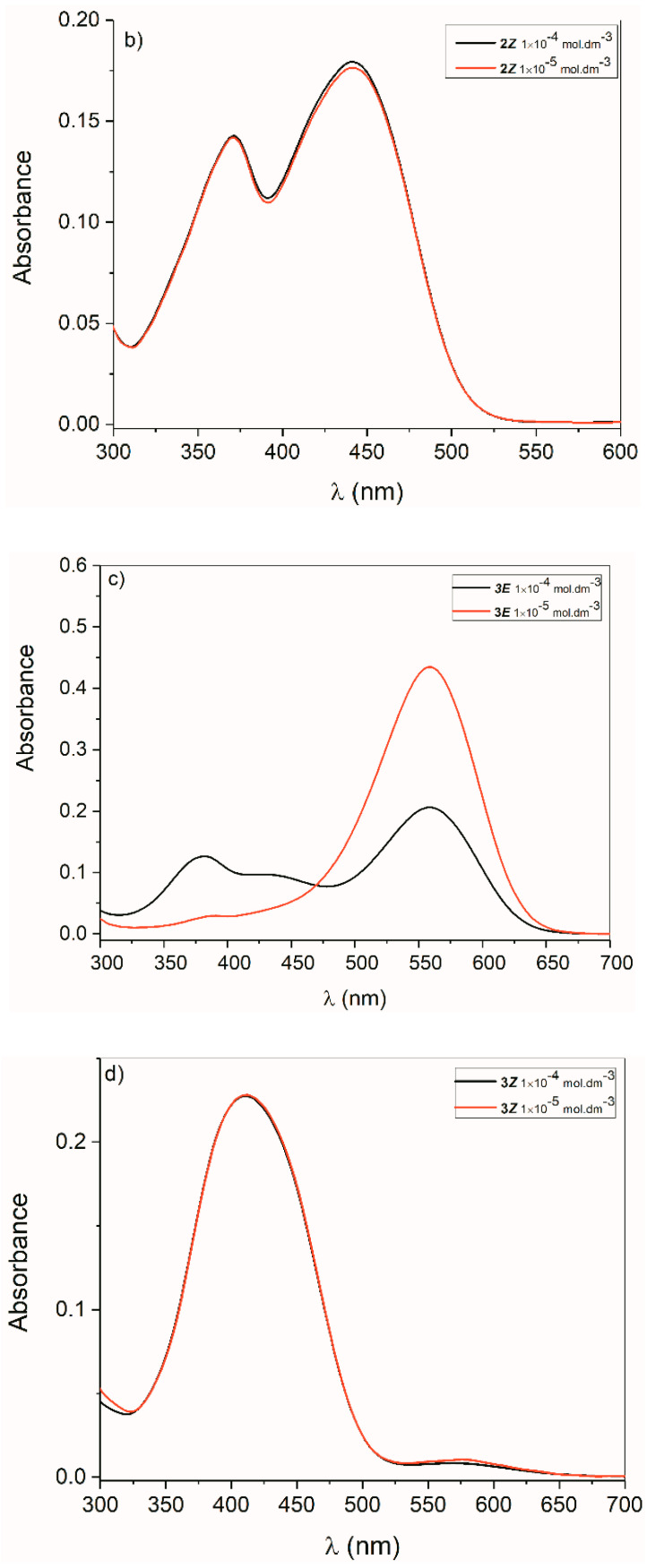
Concentration dependence of UV-Vis spectra of hydrazone *E*- and *Z*-isomers: (**a**) **2 *E***; (**b**) **2 *Z***; (**c**) **3 *E***; (**d**) **3 *Z*** in DMF (recalculated to concentration 1 × 10^−5^ mol·dm^−3^).

UV-Vis spectra of *E*- and *Z*-isomers of hydrazones **1**, **2 *E***, **2 *Z* 3 *E*** and **3 Z**, are dependent on their concentration (Figure 2; Supplementary Figure S1).

As the concentration of *E*-isomers of hydrazones with electron acceptor aryl (e.g., isatin nitrophenylhydrazones **2*E*** and **3*E***) decreases in DMF, the intensity of the absorption band increases, with a maximum around 550 nm. At the same time, the intensity of absorption around 400 nm decreases. The same changes with the change in concentration were observed in the case of isatin naphthylimide hydrazones [11] and isatin 4-nitrophenyl-hydrazone [12].

**Scheme 3:**
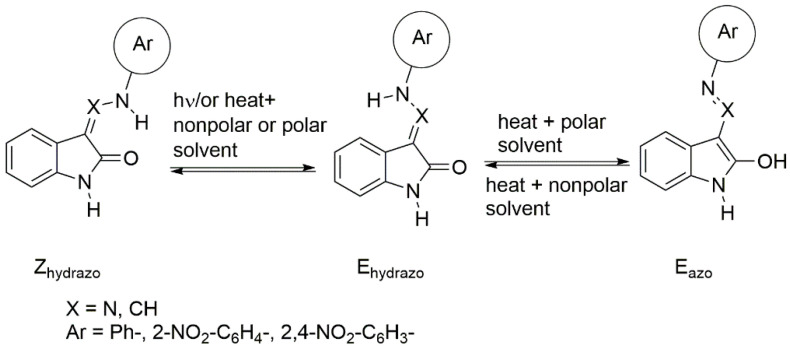
The behavior of studied compounds under different conditions.

Unlike the *E*-isomers, the *Z*-isomers did not have an absorption band in the UV-Vis spectrum with a maximum around 550 nm. The absorption band intensity of the *Z*-isomers with a maximum at 400 nm changed only slightly with decreasing concentration (Figure 2b,d; Supplementary Figure S1). The described changes in UV-Vis spectra with the change in hydrazone concentration are due to intermolecular interactions of hydrazone molecules. Intermolecular interactions affect the equilibrium state of tautomeric equilibria ***E***_hydrazo_ ↔ ***E***_azo_. As the concentration of the studied compounds increases, their molecules aggregate in the solution. Aggregation shifts the tautomeric equilibrium towards the ***E***_hydrazo_ forms. Dilution of the *E*-isomer solution to a concentration <10^−5^ mol·dm^−3^ aggregation disappears and the *E*-isomer hydrazoform is transformed into the azoform ***E_azo_*** (Scheme 3).

The *E*-isomer azo form of the studied hydrazones had a characteristic absorption band in the UV-Vis spectra with a maximum in the range from 470 nm to 600 nm (Figure 2a,c and Supplementary Figure S2b).

**Scheme 4:**
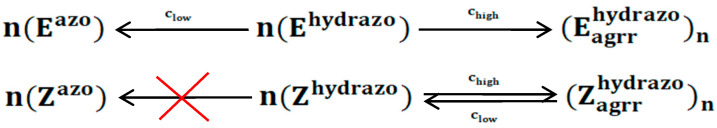
The effect of the *E*- and *Z*-isomers concentration of hydrazones on the hydrazo ↔ azo equilibrium.

**Figure 3:**
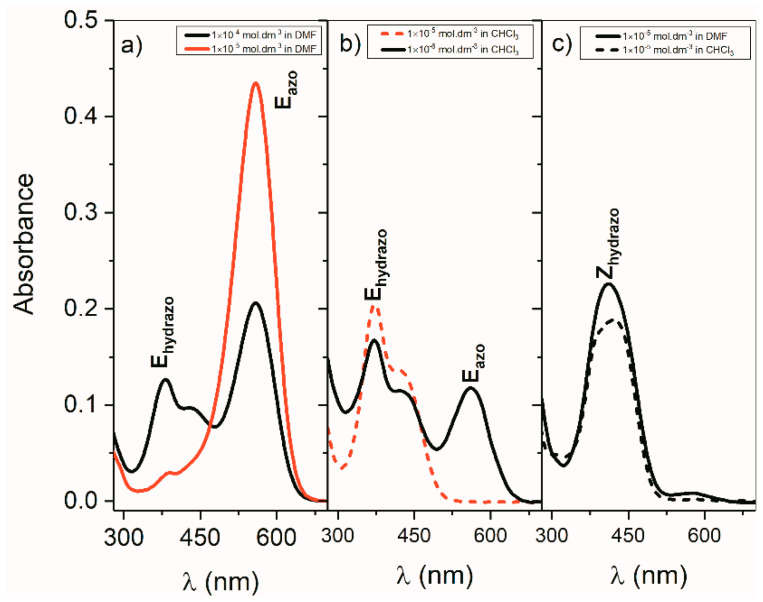
UV-Vis spectrum of hydrazone **3** at various concentrations: (**a**) **3 *E*** in DMF, (**b**) **3 *E*** in CHCl_3_, (**c**) **3 *Z*** in DMF and CHCl_3_.

The intramolecular hydrogen bond contributes to the thermodynamic stability of *Z*-isomers, which is enough to ensure that the *Z*-isomer does not tautomerize at low concentrations (no longer aggregating, Scheme 4) and room temperature (Figure 3c). The *Z*-isomer is still in the ***Z***_hydrazo_ form. The effect of the *E*- and *Z*-isomers concentration of hydrazones **2*E***, **2*Z***, **3*E***, and **3*Z*** on hydrazo ↔ azo equilibrium can be schematically represented as shown in Scheme 4.

The hydrazo ↔ azo tautomeric equilibrium of isatin arylhydrazones is also affected by their structure. E.g. the phenyl-bonded nitro group (**3*E*** and **3*Z***) by its -I inductive effect and the resonance +R electron effect stabilizes the ***E_azo_*** form and shifts the tautomeric equilibrium towards the ***E_azo_*** form. The -I effect of the substituents on the aryl itself has little effect on tautomeric equilibrium [13].

The rate of ***E_azo_*** tautomeric form formation from the *E*-isomer is high. The half-life of this reaction was t_1/2_ < 1 s. Intramolecular and intermolecular interactions contribute to the thermodynamic stability of the *E*- and *Z*-isomers of isatin arylhydrazones. It is therefore not surprising that the dielectric constant and the protic properties of the solvent affect hydrazo ↔ azo equilibrium. As an example, the effect of solvent (DMF and CHCl_3_) on the hydrazo ↔ azo equilibrium of *E*- and *Z*-isomers of hydrazone **4** (Figure 3 and Supplementary Figure S3) was shown. The polarity of the solvent (CHCl_3_, DMF) did not affect the ***Z***_hydrazo_ ↔ ***Z***_azo_ equilibrium. The *Z* isomers, both at a concentration of 1 × 10^−5^ mol·dm^−3^ in DMF and CHCl_3_, were present only in the ***Z***_hydrazo_ form (Figure 3c). Weak absorption with a maximum at 580 nm in DMF is due to the *E*-isomer presence in the solution resulting from photochemical isomerization during sample preparation for measurement. The equilibrium ***E***_hydrazo_ ↔ ***E***_azo_ in CHCl_3_ at a concentration 1 × 10^−5^ mol·dm^−3^ is completely shifted towards the ***E***_hydrazo_ form (Figure 3b). Conversely, in DMF at the same hydrazone concentration, the tautomeric equilibrium is shifted almost entirely in favor of the ***E***_azo_ form (Figure 3a).

#### 2.2.2. ((Arylamino)Methylene)Indolin-2-Ones 4 and 5

UV-Vis spectra of the *Z*-isomers (compounds **4** and **5**) are like the UV-Vis spectra of isatin arylhydrazones **1** and **3**. Compound **4** compared to **1** has a maximum long-wavelength absorption band of the ***Z_hydrazo_*** form shifted hypsochromically by 22 nm. The structural fragments =CH-NH- and =N-NH- affect the aggregation of ((arylamino)methylene)indolin-2-ones and isatin arylhydrazones. For the *Z*-isomers of compounds **4** and **5**, we observed a very small change in the UV-Vis spectra with a change in the concentration (Supplementary Figure S4). The change is so small that it cannot be attributed with any confidence to the aggregation of compounds **4** and **5**.

### 2.3. Photochemistry of Studied Compounds

#### 2.3.1. Photoisomerization of Isatin Arylhydrazones

**Figure 4:**
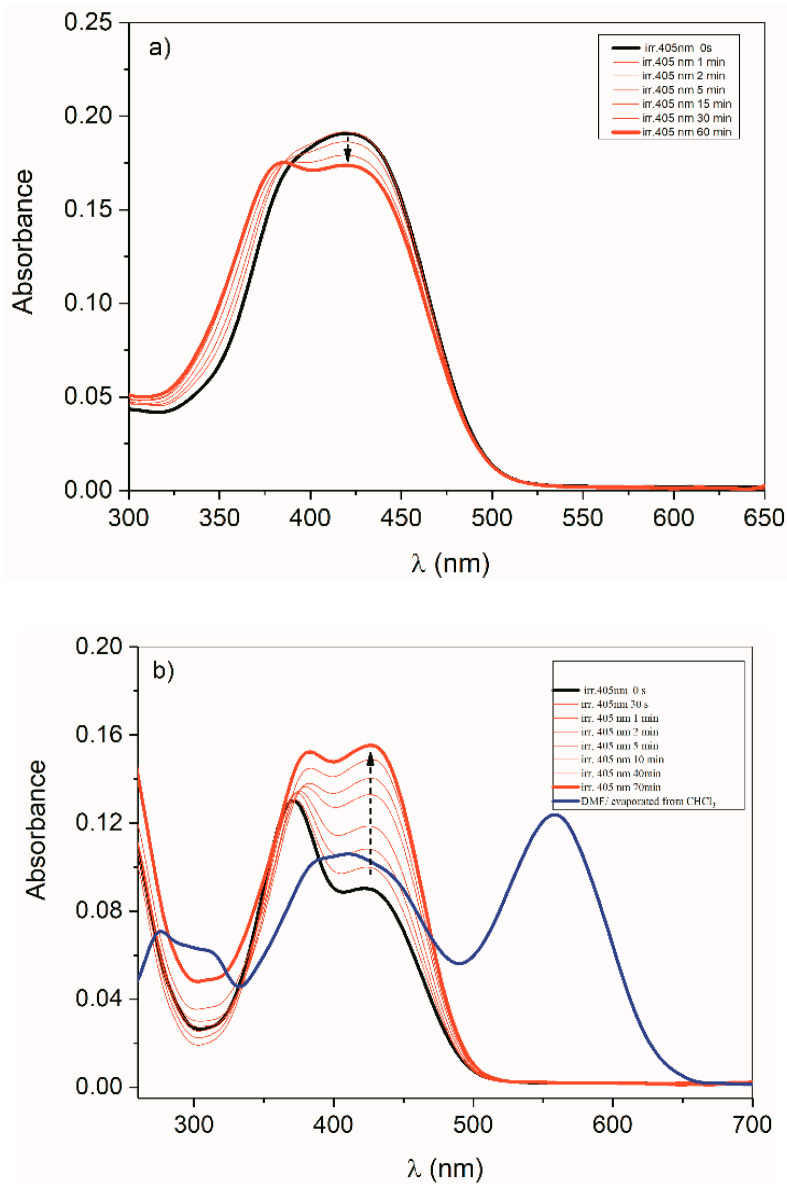
Photoisomerization of hydrazone **3** *E*- and *Z*-isomer (1 × 10^−5^ mol·dm^−3^) in CHCl_3_: (**a**) *Z*-isomer; (**b**) *E*-isomer.

The described effect of the hydrazone concentration and the solvent effect on aggregation and tautomeric equilibrium was also reflected in the photochemistry of these hydrazones. For hydrazones **1**, **2*E***, **2*Z***, **3*E***, and **3*Z***, in chloroform, as a weakly interacting solvent, the photoisomerization was the dominant photochemical reaction (Scheme 3). In UV-Vis spectra during irradiation in CHCl_3_, the intensity of the lowest energy band of *E*- or *Z*-isomers decreased, and a new absorption band was formed which was shifted hypsochromically during irradiation of *Z*-isomer and batochromically during irradiation of *E*-isomer (Figure 4).

Irradiation of the *Z*-isomer of a nitro substituted isatin arylhydrazones **2*Z*** and **3*Z*** in DMF also resulted in *Z* ↔ *E* isomerization (Table 1) as in CHCl_3_, but the resulting *E*-isomer rapidly tautomerized to the ***E_azo_*** tautomer (Scheme 3). From Table 1 and Supplementary Figures S2 and S5 is evident that the *Z*-isomers of the isatin phenylhydrazone **1** and compound **4** in DMF isomerize during irradiation but the resulting *E*-isomer does not tautomerize. *E*-isomers of isatin nitroarylhydrazones were thermally rapidly transformed into the ***E_azo_*** form.

#### 2.3.2. Phototautomerization of Isatin Arylhydrazones

**Figure 5:**
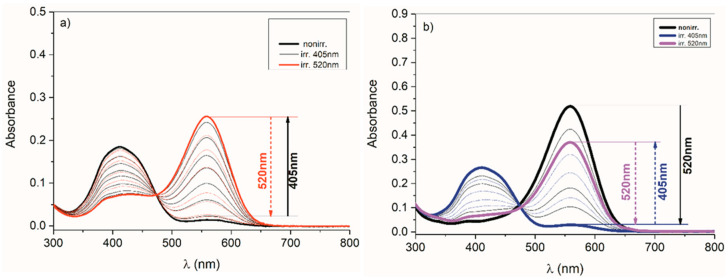
Reversible phototautomerization of hydrazone **3** (1 × 10^−5^ mol·dm^−3^) in DMF: (**a**) *Z*-isomer; (**b**) *E*-isomer.

Irradiation of a 1 × 10^−5^ mol·dm^−3^ solution of the hydrazone isomers **2*Z*** and **3*Z***, (405 nm) in DMF gave the corresponding *E*-isomer, which tautomerized very rapidly to the ***E_azo_*** tautomer (Figure 5a). For hydrazone **2 Z**, its conversion to ***E_azo_*** tautomer was very low (Supplementary Figure S2b). The resonance effect of the 4-nitro group affects the charge transfer in the hydrazone molecule to a much greater extent than the nitro group in position 2. The charge redistribution in the hydrazone molecule affects the tautomeric equilibrium. In the process of heat-stimulated tautomerization, the rate-determining step was the formation of the *E*-isomer.

**Scheme 5:**
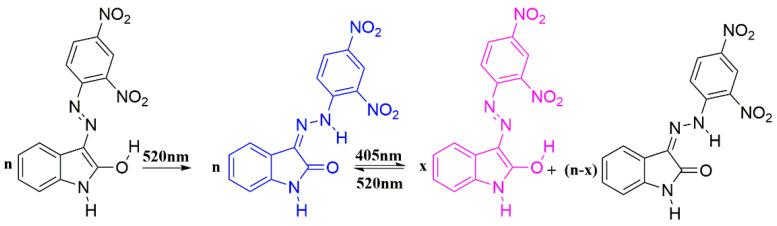
Phototautomerization of hydrazone **3**.

The of *Z* ↔ *E* isomerization rate was 10 to 100 times slower than the tautomerization rate. The ***E_azo_*** form cannot be determined directly by HPLC, because after loading the sample onto the column, the ***E_azo_*** form immediately changes to the ***E_hydrazo_*** form. At the detection wavelengths higher than 570 nm (absorption of the ***E_azo_*** form), no band was detected in the chromatogram. Therefore, the amount of ***E_azo_*** form in the sample in the chromatogram is equal to the concentrations that correspond to the ***E_hydrazo_*** form. On an example of the isatin nitrohydrazone **3**, it was demonstrated that the *E*-isomer at concentrations <1 × 10^−5^ mol·dm^−3^ was in the ***E_azo_*** form (Figure 1 and Supplementary Figure S3). Irradiation of the ***E_azo_*** form with 520 nm light produced the isomer hydrazo form (Figure 5b). Subsequent irradiation of the reaction mixture with 405 nm light of the hydrazo form of *Z*-isomer re-forms the ***E_azo_*** form. After this irradiation, the absorption band with a maximum at 560 nm (***E_azo_*** form) in the photostationary state no longer reaches the intensity it had before its first irradiation with 520 nm light. Its intensity was approximately 1/3 less (Figure 5b). Upon further alternating irradiation of the reaction mixture with 405 nm and 520 nm light, the reversible phototautomerization of ***E_azo_**** = **Z_hydrazo_*** occurred. The approximately one-third difference in the absorption band intensity with a maximum at 560 nm, which occurred between the first and second irradiation cycles, was due to the 405 nm irradiation achieving equilibrium between ***E_azo_*** and ***Z_hydrazo_*** forms (Scheme 5). Before the first irradiation (520 nm) only the *E*-isomer (1 × 10^−5^ mol·dm^−3^) in the reaction mixture was present.

For all nitro-substituted isatin arylhydrazones the same course was observed. The *E*-isomer of isatin *p*-tolylhydrazone does not produce the ***E_azo_*** tautomeric form, even in DMF, neither thermally nor photochemically (Supplementary Figure S2).

#### 2.3.3. Photoisomerisation of ((Arylamino)Methylene)Indolin-2-Ones 4 and 5

Similarly, to the *Z*-isomers of isatin arylhydrazones, compound **4** isomerized in both polar and non-polar solvents during irradiation (Supplementary Figure S5a). In the case of compound **4**-probably due to rapid intramolecular hydrogen transfer in an excited state or efficient ISC electron transition-*E*-isomer was produced in very small quantities (Supplementary Figure S5b, Table 1). These isomerizations are reversible photochemical reactions. The changes observed on the UV-Vis spectra during the irradiation of compound **5** and the ratio of *Z*/*E* isomers in the photostationary reaction mixture was like that observed during the isatin *p*-tolylhydrazone **1** irradiation (Table 1).

#### 2.3.4. Phototautomerization of ((Arylamino)Methylene)Indolin-2-Ones 4 and 5

The *Z* and *E*-isomers of compound **4** did not tautomerize in either CHCl_3_ or DMF (Scheme 5). After irradiation (a mixture of *E*- and *Z*-isomers, Table 1), absorption in the range from 470 nm to 600 nm (Supplementary Figure S5) characteristic for the ***E_azo_*** form in UV-Vis spectra was not observed. The ***E_azo_*** form was not formed similarly to the isatin *p*-tolylhydrazone **1**. In the case of compound **5**, we were unable to demonstrate whether the *E*-isomer undergoes tautomerization as isatin nitroarylhydrazones. Irradiation of compound **5** in DMF generates such a small amount of the *E*-isomer that it is not possible to confirm unambiguously the presence of the tautomeric form in the photostationary mixture by UV-Vis spectroscopy (Supplementary Figure S5, Table 1).

### 2.4. Anions of Studied Compounds

**Scheme 6:**
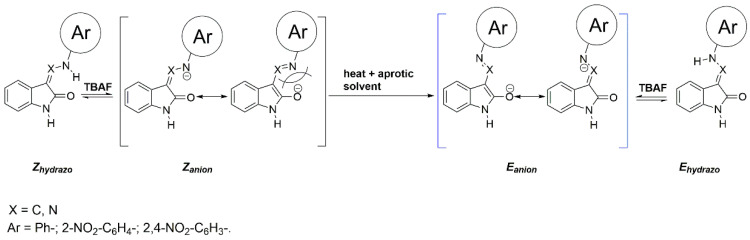
Formation and transformation of anions of studied compounds.

The nature of the aryl group in isatin arylhydrazones affects the acid-base properties of the NH hydrogens of these compounds. Although the chemical shift of NH hydrogen may not directly correlate with the NH hydrogen acid-base properties, its value is influenced by the pK values of these compounds. In the ^1^H-NMR spectra, the NH hydrogen signals have chemical shift values δ 14 to 10 ppm. *E*-isomers of hydrazones have chemical shift values of NH hydrogen at a higher field (δ = 12.5 to 10 ppm) than *Z*-isomers. The acidic nature of the NH hydrogens of isatin arylhydrazones allows us to generate hydrazone anions using a suitable base. By selecting the base and the hydrazone structure, one can specifically generate either the anion of the hydrazone formed by the cleavage of NH hydrogen from the hydrazone moiety of the molecule, or the cleavage of NH hydrogen from isatin [14]. Tetrabutylammonium fluoride (TBAF) is a base capable of generating an anion by cleaving NH hydrogen from a hydrazone molecule (Scheme 6).

**Figure 6:**
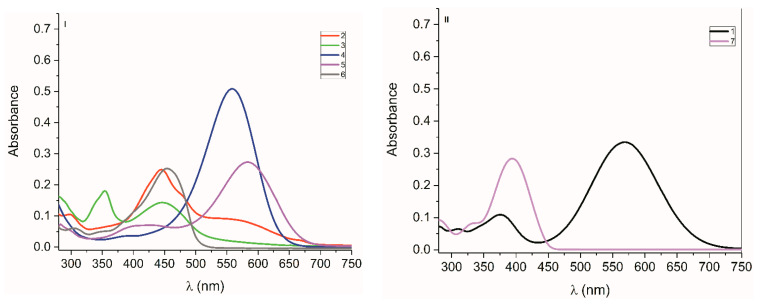
UV-Vis spectra of anions (1 × 10^−5^ mol·dm^−3^) in the presence of TBAF (5 × 10^−4^ mol·dm^−3^) in DMF: (**I**) (2) **2 *E***, (3) **2 *Z***, (4) **3 *E*** (5) **3 *Z***, (6) **1 *Z***, (**II**) (1) **5 *Z***, (7) **4 *Z***.

The lowest energy absorption band of isatin arylhydrazones anions absorbs light in the region from 450–700 nm. If there is a NO_2_ substituent on the phenylhydrazine moiety in the para position, the charge delocalization efficiency also increases significantly in this anion molecule part. The position of the absorption band maximum of the anion was in the region 450–700 nm and depended on the substitution of phenyl (Figure 6). The extent of charge delocalization in the anion is also influenced by the replacement of the hydrazone nitrogen -N= for the -CH=. E.g. the anion of compound **5**—due to the different electronegativity of nitrogen and carbon atoms—has an absorption maximum compared to the para-nitro substituted hydrazone anion (**3 *Z***) shifted hypsochromically by 23 nm (Figure 6). For compounds **1** and **4**, the hypsochromic shift is up to 62 nm.

**Figure 7:**
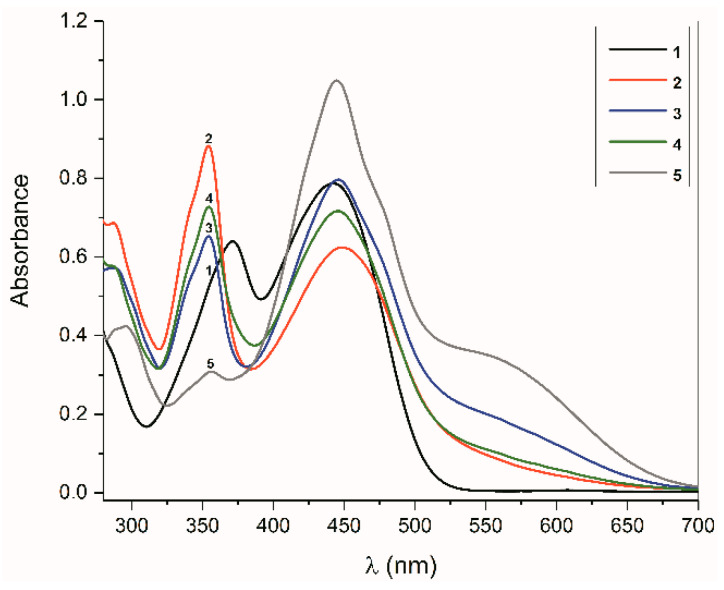
Change of UV-Vis spectrum of compound **2** (5 × 10^−5^ mol·dm^−3^), in the presence of TBAF (1 × 10^−3^ mol·dm^−3^), and during irradiation in DMF: (1) hydrazone **2**; (2) hydrazone **2** + TBAF; (3) mixture (2) after 3 h in the dark, 25 °C; (4) mixture (3) after irradiation with 430 nm light; (5) mixture (4) after 24 h in the dark 25 °C.

From the isomers of the studied compounds in the presence of TBAF, ***Z_anion_*** was formed at room temperature. ***Z_anion_*** thermally isomerized. The anion of the *E*-isomer was formed, ***E_anion_*** as a result of Coulomb repulsive forces (Scheme 6). The structure of isatin hydrazones affects the kinetics of the individual reaction steps (Scheme 6). For the studied compounds with a para nitro substituent on aryl, the acid-base reaction leading to the formation of ***Z***_anion_ was considerably faster than the subsequent reaction stage of the ***E***_anion_ formation. Therefore, the rate-determining step of the ***E***_anion_ formation process is the ***Z***_anion_-*E*_anion_ isomerization. This process was evident from UV-Vis spectra by the immediate formation of an absorption band in the wavelength range from 450 nm to 700 nm and subsequent hypsochromic shift (about 20 nm) of the band maximum (Supplementary Figure S6). The maxima hypsochromic shift confirms the existence of the ***Z***_anion_-***E***_anion_ isomerization process. The course of these processes is evident from the ^1^H-NMR spectra (Supplementary Figure S7) and HPLC records (Supplementary Figure S8). In the presence of TBAF, the NH hydrogen was not cleaved from the hydrazine part of hydrazone **2**, but NH hydrogen was cleaved from isatin. The resonance effect of the NO_2_ group in position 4 has a significantly greater effect on the charge transfer in the nitrohydrazone anion molecule than the resonant effect of the NO_2_ group in position 2. Therefore, NH hydrogen was not cleaved from the hydrazine part of 2-nitrophenylhydrazone **2** in the presence of TBAF as in the case of hydrazones having phenyl substituted with a nitro group in position 4, e.g., hydrazone **3**. The described cleavage of NH hydrogen from the hydrazone **2** in the presence of TBAF was the same for both the *Z*- and the *E*-isomer. In the corresponding UV-Vis spectra, this was reflected in the case of *Z*-isomer by a hypsochromic shift of the absorption band maximum from 373 nm to 354 nm and low absorption with a maximum at 550 nm (Figure 7).

Hydrazone **1** upon the addition of TBAF provided a ***Z*_anion,_** in which the negative charge was located on the nitrogen of the hydrazone part of the molecule. The immediate formation of this anion was observed on the ^1^H-NMR spectra by the presence of characteristic signals [14] with chemical shifts from 6.85 to 6.6; 8.1 to 8.2 and NH signals from 13 to 9 (Supplementary Figure S7). This anion has a smaller charge transfer than para nitro derivatives. Therefore, in this anion, the bathochromic shift of the band with the lowest energy was smaller than that of isatin nitroarylhydrazones. ***Z*_anion_** was already transformed into ***E*_anion_** at room temperature. The intensity of the absorption band (maximum 456 nm) increased with the increase in reaction time, without shifting of the maximum (Figure 6). In the example of hydrazone **1**, we demonstrated the effect of TBAF concentration on the kinetics of the processes described in Table 2. The dependence of the rate constant k [s^−1^] on the TBAF concentration can be described by the equation y = 0.056 − 168.92x + 212724.04 × ^2^ (Supplementary Figure S9) which confirms that the rate constant describes the process of a complex reaction scheme. The TBAF concentration may have a different effect on each of these reactions. Therefore, the measured rate constant at TBAF concentrations c_TBAF_ < 4 × 10^−4^ mol·dm^−3^ decreases with increasing TBAF concentration. At high concentrations of TBAF (>1 × 10^−2^ mol·dm^−3^) in DMF, the isomerization of ***Z***_anion_-***E***_anion_ was already so rapid that only constant absorption intensity at 450 nm—steady state— can be registered on the UV-Vis spectra.

**Scheme 7:**
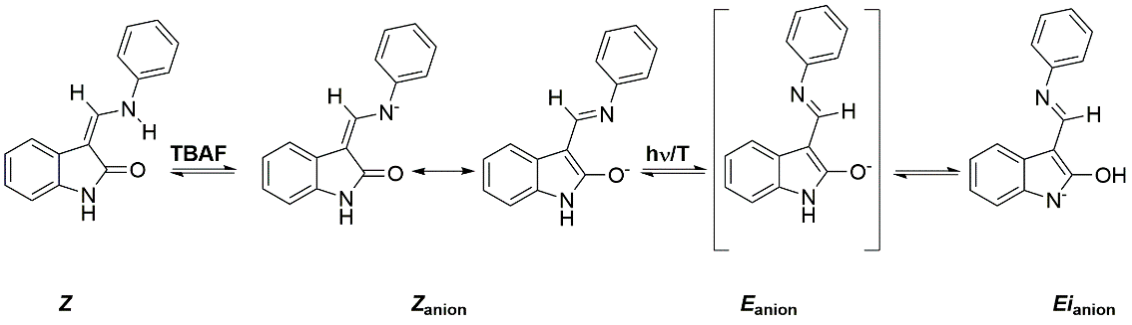
Formation and transformation of anions of compound **4**.

After the addition of TBAF to hydrazone **1**, the presence of ***E_anion_*** in solution was proved by spectral measurements and by HPLC in the ***E_hydrazo_*** form (Supplementary Figure S8). It is evident from the comparison of Supplementary Figure S8a,b and Supplementary Figure S8c,d), that the absorption with a maximum at 450 nm belongs to the ***E_anion_*** absorption band. The effect of the -NH-CH= and the -NH-N= fragment on the anion formation is evident from a comparison of compounds **1** and **4** in the presence of TBAF. After the addition of TBAF to the solution of **4**, the ***Z_anion_*** (Scheme 7) was formed by cleavage of NH hydrogen, as in the case of ***Z_anion_*** from hydrazone **1** (Scheme 6). ***Z_anion_*** formed from compound **4** had an absorption band with a maximum at 398 nm, which is shifted by 18 nm in comparison with the band of the non-ionic form (Supplementary Figures S10b,d, and S14i). ***Z_anion_*** of compounds **1** and **4** were subjected to isomerization at room temperature or by light absorption. Corresponding ***E_anions_*** of the *E*-isomers were formed. For hydrazone **1** this ***E_anion_*** was stable. ***E_anion_*** resulting from compound **4** was transformed into ***Ei_anion_*** (Scheme 7), (Supplementary Figures S10g and S11a). In the UV-Vis spectra, after transformation, a new low-intensity absorption band with a maximum at 600 nm was formed, which is characteristic for this type of ***Ei_anion_*** (Supplementary Figures S10h, S11a, and S12).

**Figure 8:**
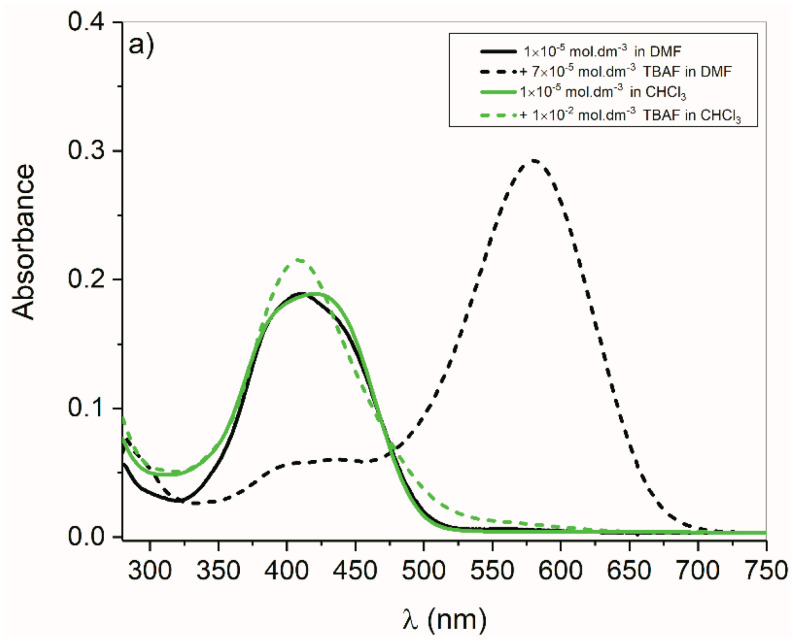

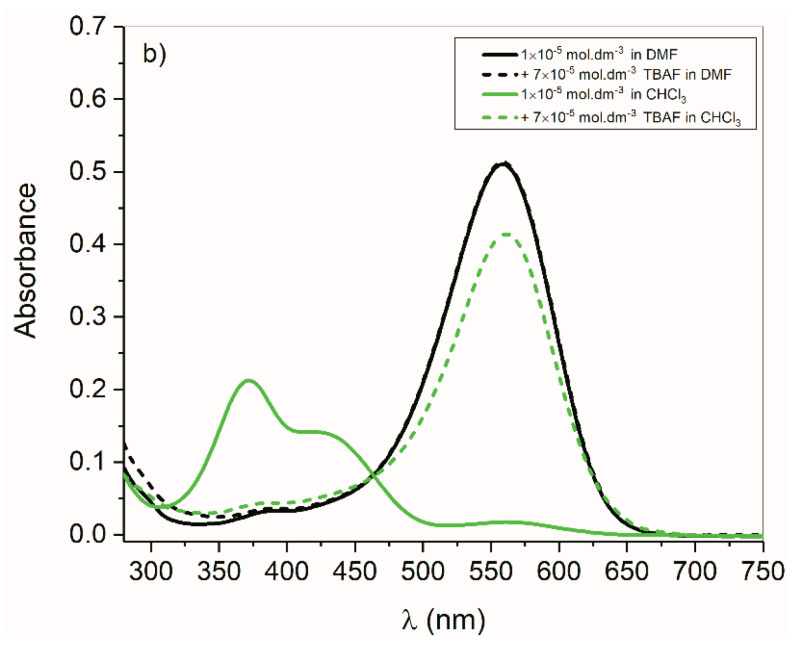
Solvent effect (DMF, CHCl_3_) on the formation of compounds **4** anions of *E-* and *Z-*isomers in the presence of TBAF: (**a**) **3 *E***; (**b**) **3 *Z***.

The ***Z_anion_***-***Ei_anion_*** isomerization rate constant at a TBAF concentration 1 × 10^−2^ mol·dm^−3^ was equal to 0.002 s^−1^ (Table 3). This value was approximately 20 times smaller as for the ***Z_anion_***-***E_anion_*** isomerization (Scheme 7) for hydrazone **1** at TBAF concentration 1 × 10^−3^ mol·dm^−3^. As it is evident from supplementary Figure S11b, the rate of ***Z_anion_*** formation of compound **5** at TBAF concentration equal 2 × 10^−4^ mol·dm^−3^ was approximately 100 times higher as the ***Z_anion_***-***E_anion_*** isomerization rate (Table 2). The nitro group in the *para* position on the aryl of compound **5** stabilizes the ***E_anion_*** with its resonance effect (Scheme 7). The equilibrium state of the ***E_anion_***-***Ei_anion_*** process was significantly shifted in favor of ***E_anion_*** (Supplementary Figure S13g). The ***E_anion_*** was characterized by an intense band with a maximum at 570 nm (Supplementary Figure S13h). The processes characterizing the anion formation of compound **5** in the presence of TBAF are the same as those of isatin nitroarylhydrazones. The *E*-isomers of the studied compounds immediately upon TBAF addition gave the corresponding ***E_anions_*** (Scheme 7). The quantification of the ***E_anion_***, ***Z_anion_*** of *E-* and *Z*-isomers, could be performed by HPLC by determining the corresponding ***E_hydrazo_*** and ***Z_hydrazo_*** isomers (not anions). The high-water content of the mobile phase ensured that, immediately after loading of the ***Z_anions_*** and ***E_anions_*** sample, the corresponding isomers were formed. The anion concentration was proportional to the concentration of the analyzed isomers (Supplementary Figure S8). The solvent effect on the anion formation can be seen in the example of hydrazone **3** (Figure 8). The solvent (DMF, CHCl_3_) had a very low effect on the formation of the ***E_anion_***, of the hydrazone **3***E*-isomer.

The *Z*-isomer of hydrazone **3** in the presence of TBAF in DMF provided ***Z_anion_***, which was transformed into the ***E_anion_*** (Scheme 7). Neither ***Z_anion_*** nor ***E_anion_*** was formed in CHCl_3_, even in the presence of a 200-fold excess of TBAF from the *Z*-isomer of **3**. The product formed in the UV-Vis spectrum did not have an intense band characteristic for these anions in the range from 450 nm to 700 nm. In the presence of TBAF, in CHCl_3_, the *Z*-isomer of hydrazone **3** weakly absorbs at 525 nm and the band maximum at 424 nm is shifted to 408 nm (Figure 8b). Such changes in the UV-Vis spectrum are characteristic for isatin aza anions and isatin hydrazone anions [14,15]. This was probably due to the higher stability of the intramolecular hydrogen bond in CHCl_3_ and the different solvation or dissociation of TBAF in DMF and CHCl_3_, respectively.

### 2.5. ON/OFF Switching

The photochemical and thermal reactivity of isatin arylhydrazones **1**–**3** and ((arylamino)methylene)indolin-2-ones **4** and **5** and the reversibility of these processes form the basic prerequisite for their possible applications as electronics materials, especially as ON/OFF signal switches. The presence of an exocyclic C=N or C=C bond of these compounds gives the possibility to generate their *E*- and *Z*-isomers by appropriate stimulation (heat, light). Reversible, stimulated change of one isomer to another can be used for ON/OFF signal switching using UV-Vis spectroscopy to monitor the ON and OFF state of the switch or logic 1 and 0 respectively. Acidic NH hydrogens are present in the structure of the studied compounds. In the presence of a suitable base, the N anions of the *E*- and *Z*-isomers are formed after cleavage of these hydrogens. The delocalization of the negative charge of these anions is reflected in the UV-Vis spectra by the formation of a new lowest energy absorption band. Depending on the anion structure, this band can be shifted by more than 100 nm from the original band of the neutral molecule. In these cases, the absorption bands of the anion and nonionic molecule do not overlap. This is an ideal case for unambiguously defining ON and OFF states.

#### 2.5.1. ON/OFF Switching of Isatin Arylhydrazones **1**–**3** and *((Arylamino)Methylene)Indolin-2-Ones* **4** and **5**

**Scheme 8:**
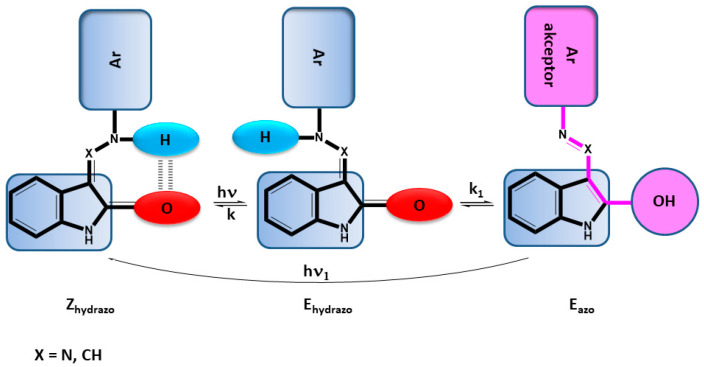
Schematic representation of studied compounds ON/OFF functionality.

Stimulation of isatin arylhydrazones with the light of a suitable wavelength results in a change in the geometry of the molecule (Scheme 3). This *Z* ↔ *E* isomerization is a reversible photochemical reaction. The excited state of the *Z*-isomer attenuates the intramolecular hydrogen bond and the hydrazine part of the molecule is rotated about an exocyclic C=N or C=C bond by 180 degrees. The thermal stability of the *Z*-isomers and the formed *E*-isomers depends on the structure of the aryl and the solvation capability of the solvent. The aryl structure of the compounds influences the thermodynamic stability of the *Z*- and *E*-isomers in various ways. The different mode of stabilization of *Z*- and *E*-isomers also affects the process of ON/OFF functionality of the studied compounds. For compounds in which aryl affects isomerization only through the rotational barrier of fragments revolving around the C=C or C=N exocyclic bonds, the ON/OFF functionality is based on ***Z*_hydrazo_** ↔ ***E*_hydrazo_** isomerization (Scheme 8).

Switching between ON and OFF states is done in both directions by light stimulation or alternately by light and heat. This group includes compounds **1** and **4** in which aryl is unsubstituted phenyl. The UV-Vis spectra of ***Z*_hydrazo_** and ***E*_hydrazo_** isomers of these compounds overlap strongly, which, in competition with other compounds, decreases their application potential as molecular switches. The ON/OFF functionality is maintained when the solvent is changed. Also, changing the structural fragment =N- to =CH- will not alter the ON/OFF functionality (Supplementary Figures S2 and S5a). Nitro substituted phenyl in the structures **2 *E***, **2** *Z*, **3 *E***, and **3 *Z*** changes the process of their ON/OFF functionality. Upon stimulation of their ***Z*_hydrazo_** isomers with light (405 nm), the ***E*_hydrazo_** isomers are formed similarly to the compounds **1** and **4**, but these are transformed very rapidly (t_1/2_ < 1 s) into the ***E*_azo_** tautomer (Scheme 6). The ***E*_azo_** tautomer forms an intramolecular hydrogen bond. Irradiation of the ***E*_azo_** tautomer of these compounds (520–590 nm) produced the ***Z*_hydrazo_** isomer (Figure 5a). It is possible to discuss when the hydrogen transfer from oxygen back to nitrogen takes place. It is highly probable that this hydrogen transfer will take place within the intramolecular hydrogen interaction after changing the geometry from the ***E*** configuration to the ***Z*** configuration. For these compounds, the ON and OFF states can be defined by the ***Z*_hydrazo_** isomer and the ***E*_azo_** tautomeric form, or vice versa. The UV-Vis spectra characterizing the ON and OFF states do not overlap, which creates very good conditions for unambiguously defining logic 1 and 0. Vis-Vis switching is another advantage. In a nonpolar weakly interacting solvent (e.g., CHCl_3_), these compounds behave similarly to **1** and **4**, when stimulated with light. There was no tautomerization of the ***E*_hydrazo_** isomer in CHCl_3_ as it was in DMF and the ON and OFF states were defined by the ***Z*_hydrazo_** and ***E*_hydrazo_** isomers (Figure 4a, Scheme 6). The ON/OFF functionality ceases by replacing the structural fragment =N- for the fragment =CH- (compound **5**). Compound **5** is photostable in both DMF and CHCl_3_ (Supplementary Figure S5b).

#### 2.5.2. ON/OFF Switching of Anions of Arylhydrazone **1**–**3** and Compounds **4** and **5**

**Scheme 9:**
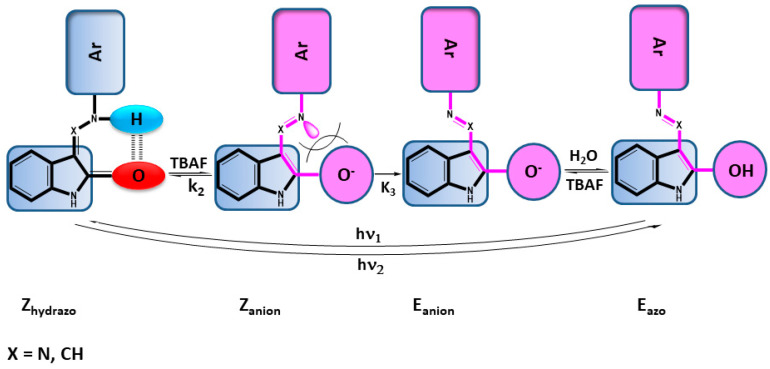
Schematic representation of studied nitro compounds ON/OFF functionality.

The *Z*-isomers of all studied compounds in the presence of TBAF formed ***Z*_anions_**, which were thermally transformed into ***E*_anions_** (Scheme 9). The electron structure of aryl has a great effect on charge transfer, especially in ***Z*_anion_**. The negative charge on the nitrogen of nitro-substituted compounds is rapidly delocalized through π bonds to oxygen after the hydrogen is removed (Scheme 9). In the UV-Vis spectra, a band with a maximum at about 550 nm appeared immediately after the TBAF addition.

**Figure 9:**
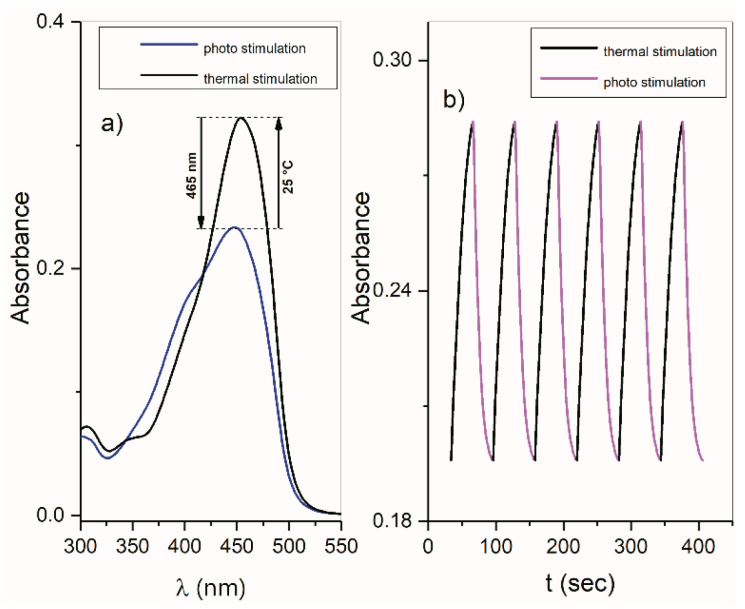
ON and OFF states of hydrazone 1 (1 × 10^−5^ mol·dm^−3^) in the presence of TBAF (5 × 10^−4^ mol·dm^−3^) at 25 °C: (**a**) UV-Vis spectra, (**b**) kinetic record—thermal and photochemical stimulation.

The phenyl of the hydrazone **1** does not allow as much charge delocalization as nitro substituted phenyl. The negative charge of these compounds was more localized on hydrazone nitrogen. The UV-Vis spectra change slightly when TBAF was added to these hydrazones (formation of ***Z*_anion_**). It was only during the transformation of ***Z*_anion_** to ***E*_anion_**, that in the UV-Vis spectrum a new band with a maximum at 456 nm was formed (Figure 9)

The equilibrium state shift towards the anionic forms depends on the concentration of TBAF. With a large excess of TBAF (>20 equivalents) in DMF, this equilibrium was almost entirely shifted in favor of the anionic forms ***Z*_anion_** and ***E*_anion_**. Under such conditions, these compounds lost the ON/OFF functionality. After anion excitation (470 nm, 520 nm, or 590 nm) there was no absorption loss in this spectral region as in the switching between ***Z*_hydrazo_** and ***E*_azo_** forms (Scheme 8).

**Figure 10:**
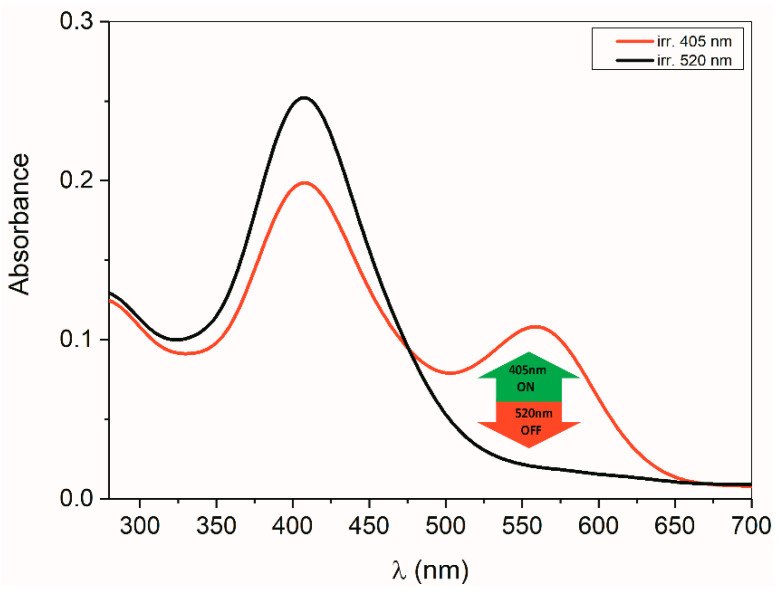
Photoswitching of anion of hydrazone **3** (1 × 10^−5^ mol·dm^−3^) + TBAF (1 × 10^−2^ mol·dm^−3^) in CHCl_3_.

The ON/OFF functionality of the studied compounds in the presence of TBAF could be observed at concentrations of F^−^ at which, enough concentration of the ***Z*_hydrazo_** form, was still present in the solution. The presence of water or a proton donor compound is also an important factor in ensuring ON/OFF functionality. The protonodonorous system shifts the equilibrium ***E*_anion_** = ***E*_azo_** in favor of ***E*_azo_** (Scheme 9). In such a system, alternating radiation (520 nm/405 nm) results in switching between ***Z*_hydrazo_** and ***E*_azo_** forms of the studied compounds (Scheme 9). Since this is switching between the same forms as in the anion-free system, (Scheme 8), it might seem, that the presence of anions is unnecessary for the ON/OFF functionality of the studied compounds. The presence of ***E*_anion_** in the switching system is a necessary condition to provide ON/OFF functionality of hydrazones **1** and compounds **4**, **5** that do not tautomerize in the absence of a base (e.g., TBAF). At these compounds, this ensures the ***E*_azo_** form presence in the system and the ability to switch between the ***Z*_hydrazo_** and ***E*_azo_** forms (Scheme 9). In CHCl_3_, where the dissociation of TBAF is even more limited than in DMF, ON/OFF functionality can be observed even at a concentration of TBAF (1 × 10^−2^mol·dm^−3^) (Figure 10). By replacing the hydrazone nitrogen =N- in the studied hydrazones with a =CH- group, ON/OFF functionality of the nitro compound **5** in the presence of TBAF does not differ from this functionality of hydrazones. A disadvantage of compounds **4** and **5** is that they hydrolyze readily. In the case of compound **4**, ***E*_anion_** transforms into ***Ei*_anion_**, which does not have the ON/OFF switch properties.

## 3. Materials and Methods

### 3.1. General Information

All chemicals used for synthesis were purchased from Sigma-Aldrich (St. Louis, MO, USA) or Fluorochem Ltd. (Derbyshire, UK). Solvents were dried and purified by standard methods prior to use. The samples were irradiated in the device’s own construction directly in the spectrometer’s cell by means of LEDs (electrical input 30 to 120 mW).

### 3.2. Synthesis

#### 3.2.1. Synthesis of Hydrazones **1**–**3**

A solution of isatin (3.4 mmol) and corresponding hydrazine (3.4 mmol) in ethanol (150 mL) was refluxed for 5 h. After cooling, the precipitate was filtered, washed with cold ethanol (3 × 10 mL), and dried. In the case of hydrazone **2*E*** the reaction mixture was stirred at room temperature for 48 h.

Hydrazone **3** was isolated as a mixture of *E*- and *Z*-isomers. Pure isomers were obtained after column chromatography on SiO_2_ using hexane:ethyl acetate (2:1) mixture as eluent.

*(Z)-3-(2-(p-Tolyl)hydrazono)indolin-2-one* (**1*Z***). Orange solid, (725 mg, 85%) ^1^H-NMR (DMSO-*d*_6_), δ: 12.76 (s, 1H), 11.00 (s, 1H), 7.54 (d, *J* = 7.4 Hz, 1H), 7.32 (d, *J* = 8.4 Hz, 2H), 7.24 (td, *J* = 7.7, 1.1 Hz, 1H), 7.18 (d, *J* = 8.3 Hz, 2H), 7.05 (td, *J* = 7.5, 0.7 Hz, 1H), 6.93 (d, *J* = 7.7 Hz, 1H), 2.28 (s, 3H). ^13^C-NMR (DMSO-*d*_6_) δ 163.21, 140.20, 132.01, 129.89, 128.20, 127.00, 121.77, 121.25, 118.42, 114.06, 110.44, 20.39. [16]

*(Z)-3-(2-(2-Nitrophenyl)hydrazono)indolin-2-one* (**2*Z***). Orange solid, (729 mg, 76%) ^1^H-NMR (DMSO-*d*_6_), δ: 14.27 (s, 1H), 11.18 (s, 1H), 8.22 (ddd, *J* = 8.5, 4.7, 1.1 Hz, 1H), 7.82 (dd, *J* = 11.5, 4.2 Hz, 1H), 7.66 (d, *J* = 7.2, 1H), 7.34 (td, *J* = 7.7, 1.2 Hz, 1H), 7.18 (ddd, *J* = 8.4, 7.1, 1.2 Hz, 1H), 7.10 (td, *J* = 7.6, 0.8 Hz, 1H), 6.95 (d, *J* = 7.8, 1H). ^13^C-NMR (DMSO-*d*_6_), δ: 163.28, 147.31, 142.53, 140.11, 137.21, 137.02, 134.36, 125.16, 125.03, 124.31, 124.02, 115.84. [17]

*(E)-3-(2-(2-Nitrophenyl)hydrazono)indolin-2-one* (**2*E***). Orange solid, (652 mg, 68%) ^1^H-NMR (DMSO-*d*_6_), δ: 11.57 (s, 1H), 10.82 (s, 1H), 8.24 (dd, *J* = 8.5, 1.3 Hz, 1H), 8.04 (dd, *J* = 8.5, 1.0 Hz, 1H), 7.91-7.79 (m, 2H), 7.44 (td, *J* = 7.7, 0.8 Hz, 1H), 7.20 (tdd, *J* = 7.5, 6.4, 1.0 Hz, 2H), 6.98 (d, *J* = 7.8, 1H). ^13^C-NMR (DMSO-*d*_6_), δ: 167.63, 146.66, 144.60, 141.70, 138.56, 138.06, 135.69, 127.41, 126.70, 125.54, 125.25, 116.07. Elem. anal. calcd. for C_14_H_10_N_4_O_3_: C 59.57, H 3.57; N 19.85. found: C 59.61; H 3.55; N 19.88. m. IR (ATR) ν/cm^−1^: 3333, 3085, 2360, 1751, 1610, 1318, 1281, 726. p.: 269–271 °C.

*(Z)-3-(2-(2,4-Dinitrophenyl)hydrazono)indolin-2-one* (**3*Z***). Orange solid, (367 mg, 33%) ^1^H-NMR (DMSO-*d*_6_), δ: 14.53 (s, 1H), 11.32 (s, 1H), 8.94 (d, *J* = 2.5 Hz, 1H), 8.55 (dd, *J* = 9.2, 2.3 Hz, 1H), 8.35 (d, *J* = 9.5 Hz, 1H), 7.71 (d, *J* = 7.4 Hz, 1H), 7.46–7.36 (td, *J* = 7.7, 1.3 Hz, 2H), 7.13 (td, *J* = 7.6, 0.9 Hz, 2H), 6.98 (d, *J* = 7.8 Hz, 1H). ^13^C-NMR (DMSO-*d*_6_) δ: 161.34, 141.72, 141.21, 137.53, 136.53, 131.52, 130.11, 129.47, 121.52, 120.35, 120.17, 114.41, 113.22, 110.17 [18]

*(E)-3-(2-(2,4-Dinitrophenyl)hydrazono)indolin-2-one* (**3*E***). Orange solid, (411 mg, 37%) ^1^H-NMR (DMSO-*d*_6_), δ: 11.75 (s, 1H), 10.95 (s, 1H), 8.93 (d, *J* = 2.6 Hz, 1H), 8.61 (dd, *J* = 9.5, 2.6 Hz, 1H), 8.16 (d, *J* = 9.5 Hz, 1H), 7.90 (d, *J* = 7.6 Hz, 1H), 7.49 (t, *J* = 7.4 Hz, 1H), 7.22 (t, *J* = 7.7 Hz, 1H), 7.00 (d, *J* = 7.7 Hz, 1H). ^13^C-NMR (DMSO-*d*_6_) δ: 164.07, 143.98, 143.85, 139.79, 138.73, 133.32, 132.24, 130.47, 123.77, 122.49, 122.17, 116.82, 115.17, 111.30. Elem. anal. calcd. for C_14_H_9_N_5_O_5_: C 51.38, H 2.77; N 21.40. found: C 51.43; H 2.81; N 21.37. IR (ATR) ν/cm^−1^: 3318, 3067, 2360, 1728, 1609, 1310, 1277, 738. m.p.: >300 °C.

#### 3.2.2. Synthesis of Compounds **5** and **6**

A solution of oxindole (500 mg, 3.75 mmol) and triethyl orthoformate (4.86 mL, 29.3 mmol) in acetic acid (10 mL) was refluxed for 1.5 h. After cooling the volume was reduced and the oily mixture was dissolved in ethanol (20 mL). The volume was reduced to half after cooling to 10 °C the precipitate was filtered off, washed with cold ethanol (2 × 10 mL), and dried under vacuo. The product was used for the next step without purification.

A solution of (ethoxymethylene)indolin-2-one (600 mg, 3 mmol) and the corresponding aniline (3 mmol), HCl (0.1 mL) in ethanol (5 mL) was refluxed for 2 h. After cooling, the precipitated solid was filtered off, washed with ethanol (10 mL), ether (10 mL), and dried under vacuo.

*(E)-3-[(Phenylamino)methylene]indolin-2-one* (**4**). Red solid (530 mg, 75%) ^1^H-NMR (DMSO-*d*_6_) δ 10.70 (d, *J* = 12.5 Hz, 1H), 10.48 (s, 1H), 8.58 (d, *J* = 12.5 Hz, 1H), 7.57 (d, *J* = 7.5 Hz, 1H), 7.38–7.34 (m, 4H), 7.08-7.03 (m, 1H), 6.99 (td, *J* = 7.6, 1.0 Hz, 1H), 6.91 (td, *J* = 7.5, 0.8 Hz, 1H), 6.83 (d, *J* = 7.6 Hz, 1H) ppm. ^13^C-NMR (DMSO-*d*_6_) δ 170.32, 140.38, 138.35, 137.39, 130.09, 124.66, 124.46, 123.48, 120.79, 117.50, 117.37, 116.28, 109.57, 100.08 ppm. IR (ATR) ν/cm^−1^: 3131, 1678, 1580, 1269, 937. [19]

*(E)-3-[((4-Nitrophenyl)amino)methylene]indolin-2-one* (**5**). Red solid (360 mg, 72%) ^1^H-NMR (DMSO-*d*_6_) δ 10.95 (d, *J* = 12.1 Hz, 1H), 10.62 (s, 1H), 8.64 (d, *J* = 12.1 Hz, 1H), 8.22 (d, *J* = 9.0 Hz, 2H), 7.60 (t, *J* = 9.1 Hz, 3H), 7.06 (t, *J* = 7.4 Hz, 1H), 6.94 (t, *J* = 7.4 Hz, 1H), 6.84 (d, *J* = 7.6 Hz, 1H). ^13^C-NMR (DMSO-*d*_6_) δ 170.18, 146.50, 142.10, 138.37, 136.21, 126.17, 126.05, 125.87, 123.95, 121.19, 118.48, 116.68, 116.13, 109.89, 103.90 ppm. Elem. anal. calcd. for C_15_H_11_N_3_O_3_: C 64.05, H 3.94; N 14.94. found: C 64.21; H 3.99; N 14.89. IR (ATR) ν/cm^−1^: 3163, 2360, 1692, 1577, 1308, 1278, 839. m.p.: >300 °C.

### 3.3. Spectroscopic Measurements

NMR spectra were recorded in 5 mm NMR tubes on a VNMRS 600 MHz spectrometer (600 MHz for ^1^H; 150 MHz for ^13^C, Varian, Inc., Palo Alto, CA, USA) in DMSO-*d*_6_ and CDCl_3_ as solvents. Chemical shifts are referenced to tetramethylsilane (TMS) as an internal standard. Attenuated total reflection Fourier transform infrared (ATR-FTIR) spectra of all the described experiments were measured on a Nicolet 6700 FTIR (from ThermoNicolet Corp., Madison, WI, USA). Spectra were recorded with ATR mathematical corrections yielding a 1.0 cm^−1^ actual resolution and 40 measurements were averaged. Electronic absorption spectra were obtained on an HP 8452A diode array spectrophotometer (Hewlett Packard, Palo Alto, CA, USA). The solvents used (CHCl_3_ and DMF) were HPLC (CHCl_3_; LiChrosolv^®^, Merck, Darmstadt, Germany) or UV-spectroscopy grade (DMF; Uvasol^®^, Merck) and were used without further purification.

### 3.4. HPLC Chromatography

HPLC chromatography was carried out using a chromatography system (Agilent Technologies, Santa Clara, CA, USA) consisting of a quaternary pump (G 1311A), variable wavelength detector VWD G 1314A), manual injector (Rheodyne model 7725i) with 10 μL sample loop, and a degasser (g1379A) all the 1100 series.) For analyses of compounds **1** and **3**, column ZORBAX SB- Phenyl (150 mm × 4.6 mm i.d), mobile phase A water, mobile phase B 80% CH_3_OH, at the flow rate of 1 mL min^−1^ and detection at 400 nm, compounds **4** and **5** mobile phase A 30% water, mobile phase B 70% CH_3_OH, at the flow rate of 1 mL min^−1^ and detection at 400 nm was used. For analyses of compound **3** a ZORBAX SB- Phenyl (150 mm × 4.6 mm i.d) column, mobile phase A 42% water, mobile phase B 58% CH_3_CN, at ta flow rate of 1 mL·min^−1^ and detection at 390 nm was used.

## 4. Conclusions

The photochemical reactivity of isatin arylhydrazones is dependent on the structure of the aryl and on the environment in which the photochemical reaction takes place. Aggregates form at a concentration higher than 1 × 10^−5^ mol·dm^−3^. The *Z-* and *E-*isomers of isatin phenylhydrazones isomerize after light absorption in CHCl_3_ and DMF. Nitro-substituted isatin arylhydrazones also isomerize in CHCl_3_. In DMF, the *E***-**isomers of a nitro substituted isatin arylhydrazones are in equilibrium with their tautomeric forms. The equilibrium state of the tautomeric reaction ***E*_hydrazo_** ↔ ***E*_azo_** is dependent on the hydrazone concentration. As the concentration of isatin nitroarylhydrazone, decreases, the concentration of the tautomeric form increases. The *Z*-isomers of these isatin nitroarylhydrazones does not tautomerise under the same conditions. Irradiation of the *Z***-**isomers in DMF provided the *E*-isomer from which the tautomeric form of ***E*_azo_** is rapidly formed. The tautomeric form of ***E*_azo_** was not transformed by irradiation into the ***E*_hydrazo_** form, but the product of this photochemical reaction was the **Z_hydrazo_** form. This process was reversible. The substitution of the =N- structural fragment by the =CH- fragment (compounds **4** and **5**) causes, that the nitro derivative **5** did not tautomerize in DMF, as isatin nitroarylhydrazones. During the irradiation of compound **5** in DMF or CHCl_3_, photochemical *Z=E* isomerization was like that of isatin phenylhydrazone. The *Z***-**isomer of **5** hardly isomerizes under the same conditions. For the studied isatin arylhydrazones **1**-**3** and ((arylamino)methylene)indolin-2-ones **4** and **5**, anions were formed by NH hydrogen cleavage from their molecule in the presence of a base (e.g., TBAF, TBAA). The anions were stable at room temperature. The new charge distribution in these anions affects their reactivity. The disruption of the intramolecular hydrogen bond and Coulomb repulsion forces between the centers of the negative charge in the *Z***-**isomers anions supports the formation of the *E***-**isomers of the anions. The ***Z*_anion_** ↔ ***E*_anion_** reaction equilibrium state is dependent on the base concentration. ***Z*_anion_** and ***E*_anion_** in the presence of a base were formed in all studied isatin arylhydrazones and ((arylamino)methylene)indolin-2-ones **4** and **5**. =N- and =CH- structural fragments do not prevent the formation of anions. The ***E*_anion_** of compound **5** was unstable and was further transformed into the anion of the *E***-**isomer, which had a negative charge located on the isatin nitrogen. The photoisomerization and tautomerization of studied compounds were reactions in which the UV-Vis spectra of the initial and final states were identified with the ON and OFF states of the signal switch. For compounds **1** and **4**, the ON and OFF states were derived from the UV-Vis spectra of the *Z-* and *E-*isomers. The reversible ***Z*_hydrazo_** ↔ ***E*_azo_** process was used to switch the signal of isatin nitroarylhydrazones. The isomerization and the tautomeric reaction were stimulated by light. Compound **5** did not have ON/OFF functionality under these conditions. For the studied compounds anions, the same equilibrium reaction ***Z*_hydrazo_** ↔ ***E*_azo_** for nonionic forms of isatin nitroarylhydrazones was used to switch between ON and OFF states. Anion generation achieves ON/OFF functionality even for compounds that did not have this functionality in a non-ionic form (e.g., **1** and **4**). ***E*_anion_** of compound **4** was transformed photochemically and thermally to ***E*_i-anion_**, which did not have ON/OFF functionality.

## Figures and Tables

**Table 1:** The concentration of *Z*-isomers (**c*z*_hydrazo_**), *E*-isomers (**c*_E_*_hydrazo_**), and (**c*_E_*_azo_**) of studied compounds in the photostationary state of *Z* ↔ *E* isomerization (λ_irr_ = 405 nm) at 25 °C in DMF.

Compound	c*_Z_*_hydrazo_ × 10^6^ [mol·dm^−3^]	c*_E_*_hydrazo_ × 10^6^ [mol·dm^−3^]	c*_Z_*_hydrazo_/c*_E_*_hydrazo_	c*_E_*_azo_
**1**	7.30	2.70	2.70	-
**2*Z***	6.64	3.36	1.98	-
**3*Z***	1.68	8.31	-	0.20
**4**	5.68	4.32	1.31	-
**5**	9.06	0.94	9.67	-

**Table 2:** Change of the isomerization rate constant k [s^−1^] ***Z_anion_*** = ***E_anion_*** of compound **1** (1 × 10^−5^ mol·dm^−3^) depending on the concentration of TBAF in DMF (25 °C).

c_TBAF_ [mol·dm^−3^]	k [s^−1^]
1 × 10^−4^	0.008
2.5 × 10^−4^	0.009
5 × 10^−4^	0.025
1 × 10^−3^	0.040

**Table 3:** ***Z_anion_*** formation rate constants **k*_Z_*_anion_** [s^−1^] and ***Z_anion_* = *E_anion_*** thermal isomerization rate constants ***k_Zanion-Eanion_*** [s^−1^] for the reaction of compounds **4** and **5** with TBAF in DMF at 25 °C.

Compound	C_TBAF_ [mol·dm^−3^]	k_Zanion_ [s^−1^]	k_Zanion-Eanion_ [s^−1^]
**4**	1 × 10^−2^	/	0.002
**5**	2 × 10^−4^	1.025	0.019

## Supplementary Materials

The following are available online, Figure S1: Concentration effect on UV-Vis spectra of *E*- and *Z*-isomers of hydrazones **1 *Z***, **2 *E***, **2 *Z***, **3 *E***, **3 *Z***, in CH_3_CN and CHCl_3_.; Figure S2: Change in UV-Vis spectra of hydrazones after irradiation with 405 nm light (a) hydrazone **1** and (b) hydrazone **2 *Z*** in DMF.; Figure S3: Effect of DMF and CHCl_3_ on hydrazo=azo equilibrium of *E*- and *Z*-isomers of hydrazone **3** (1 × 10^−5^ mol·dm^−3^).; Figure S4: Concentration effect of compounds **4** and **5** on UV-Vis spectrum in DMF.; Figure S5: Photoisomerization of (a) compound **4** (b) compound **5** in DMF (1 × 10^−5^ mol·dm^−3^).; Figure S6: UV-Vis spectra change of hydrazone **3** (1 × 10^−5^ mol·dm^−3^) depending on time at 25 °C in CHCl_3_.; Figure S7: ^1^H NMR spectrum of hydrazone **1** (a) hydrazone **1** (1 × 10^−3^ mol·dm^−3^); (b) mixture a) + TBAF (5 × 10^−5^ mol·dm^−4^); (c) mixture (b) after 20 h in the dark; (d) mixture (c) after irradiation with 405 nm light; (e) mixture (d) after irradiation with 445 nm light.; Figure S8: HPLC chromatogram and UV-Vis spectrum in DMF: (a) and (b) hydrazone **1** (5 × 10^−5^ mol·dm^−3^); (c) and (d) hydrazone **1** + TBAF (3 × 10^−4^ mol·dm^−3^); (e) and (f) mixture (c) after irradiation with 470 nm light; (g) and (h) mixture (e) after 16 h in the dark 25 °C; Figure S9: The rate constant k [s^−1^] dependence of the process *Z*_anion_=*E*_anion_ isomerization of hydrazone **1** anion (1 × 10^−5^ mol·dm^−3^) on the concentration of TBAF in DMF at 25 °C; Figure S10: Photochemistry of compound **4** (1 × 10^−3^ mol·dm^−3^) + TBAF (1 × 10^−2^ mol·dm^−3^) monitored by ^1^H NMR and UV-Vis spectra in DMSO.; Figure S11: Change of UV-Vis spectra of compounds **4** and **5** with TBAF depending on reaction time 25 °C in DMF (a) compound **4** (1 × 10^−5^ mol·dm^−3^) + TBAF (1 × 10^−2^ mol·dm^−3^); (b) **5** (1 × 10^−5^ mol·dm^−3^) + TBAF (1 × 10^−4^ mol·dm^−3^).; Figure S12: UV-Vis of compound **4** in DMF: 1—(**4** (5 × 10^−5^ mol·dm^−3^)); 2—(**4** + TBAF (5 × 10^−3^ mol·dm^−3^)); 3—(**4** + TBAF (5 × 10^−3^ mol·dm^−3^) after irradiation with 405 nm light).; Figure S13: Effect of temperature and irradiation (405 nm) on compound **5** (5 × 10^−3^ mol·dm^−3^) in the TBAF presence (5 × 10^−2^ mol·dm^−3^) monitored by ^1^H NMR and UV-Vis: (a), (b) compound **5**; (c), (d) **5** + TBAF; (e), (f) mixture (c) after 3 h at 80 °C; mixture e) after irradiation with 405 nm light.; Figure S14: Titration of *E*- and *Z*-isomers of hydrazone: (a) **3*E*** in DMF; (b) **3*Z*** in DMF; (c) **3*E*** in CH_3_CN; (d) **3*Z*** in CH_3_CN; (e) **3*E*** in CHCl_3_; (f) **3*Z*** in CHCl_3_; (g) **2*E*** in DMF; (h) **2*Z*** in DMF; (i) **1** in DMF; (j) **4** in DMF.
