# Supplementary material for: Effect of a =X-NH-Fragment, (X = C, N), on Z/E Isomerization and ON/OFF Functionality of Isatin Arylhydrazones, ((Arylamino)Methylene)Indolin-2-Ones and Their Anions
Source: Molecules. 2020 Jul 6;25(13):3082. doi: 10.3390/molecules25133082 (PMC7412119; doi:10.3390/molecules25133082)
Supplement: Supplementary file 1 [file molecules-25-03082-s001.pdf]

Effect of a =X-NH-Fragment, (X = C, N), on *Z*/*E* Isomerization and ON/OFF Functionality of Isatin Arylhydrazones, ((Arylamino)Methylene)Indolin-2-Ones and Their Anions

Pavol Tisovský *, Klaudia Csicsai, Jana Donovalová, Róbert Šandrik, Róbert Sokolík and Anton Gáplovský

Faculty of Natural Sciences, Institute of Chemistry, Comenius University, Ilkovičova 6, Mlynská dolina CH-2, SK-842 15 Bratislava, Slovakia; klaudia.jakusova@uniba.sk (K.C.); jana.donovalova@uniba.sk (J.D.); sandrik2@uniba.sk (R.Š.); robert.sokolik@uniba.sk (R.S.); anton.gaplovsky@rec.uniba.sk (A.G.)

* Correspondence: pavol.tisovsky@uniba.sk; Tel.: +421-290-149-378

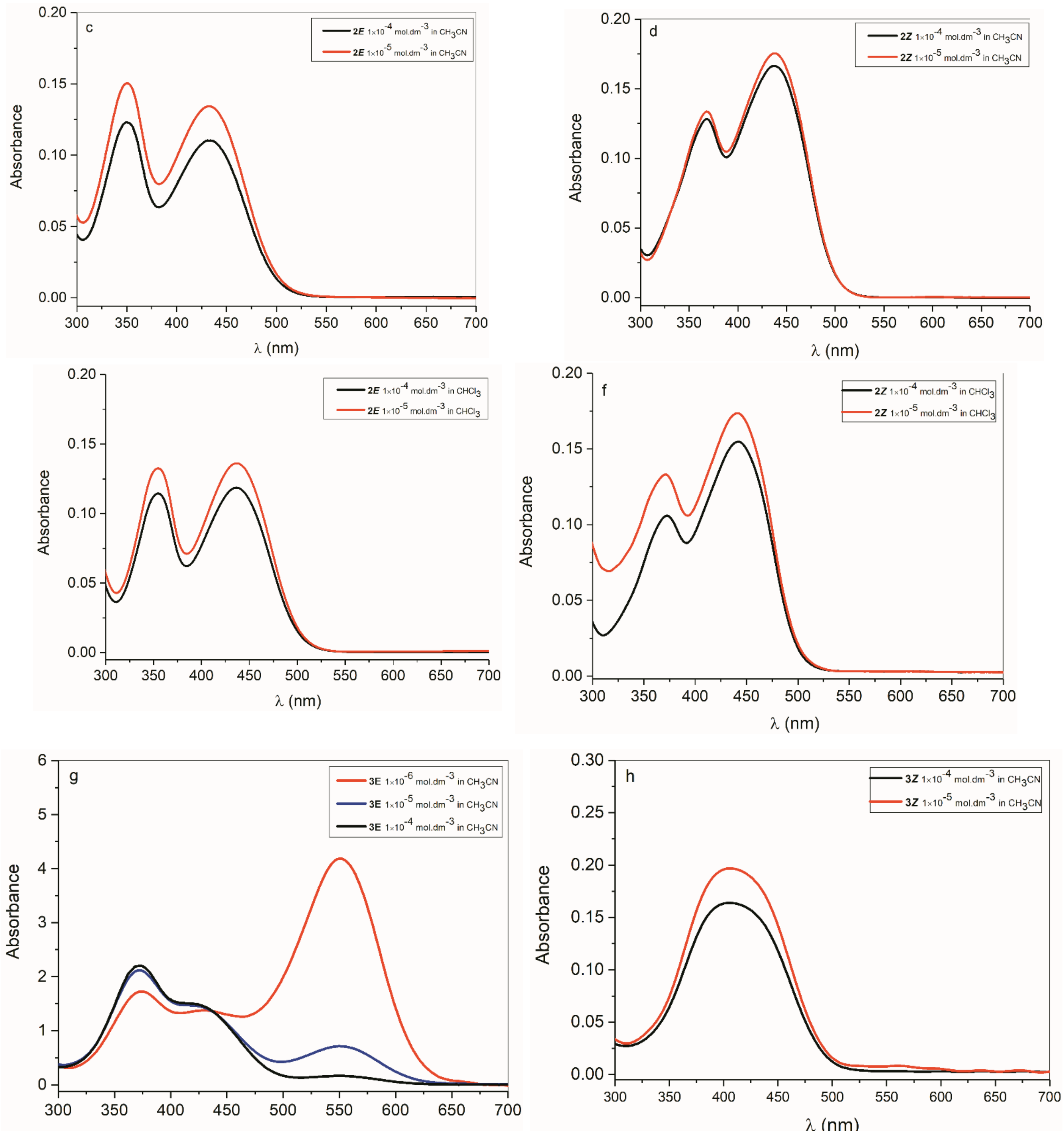

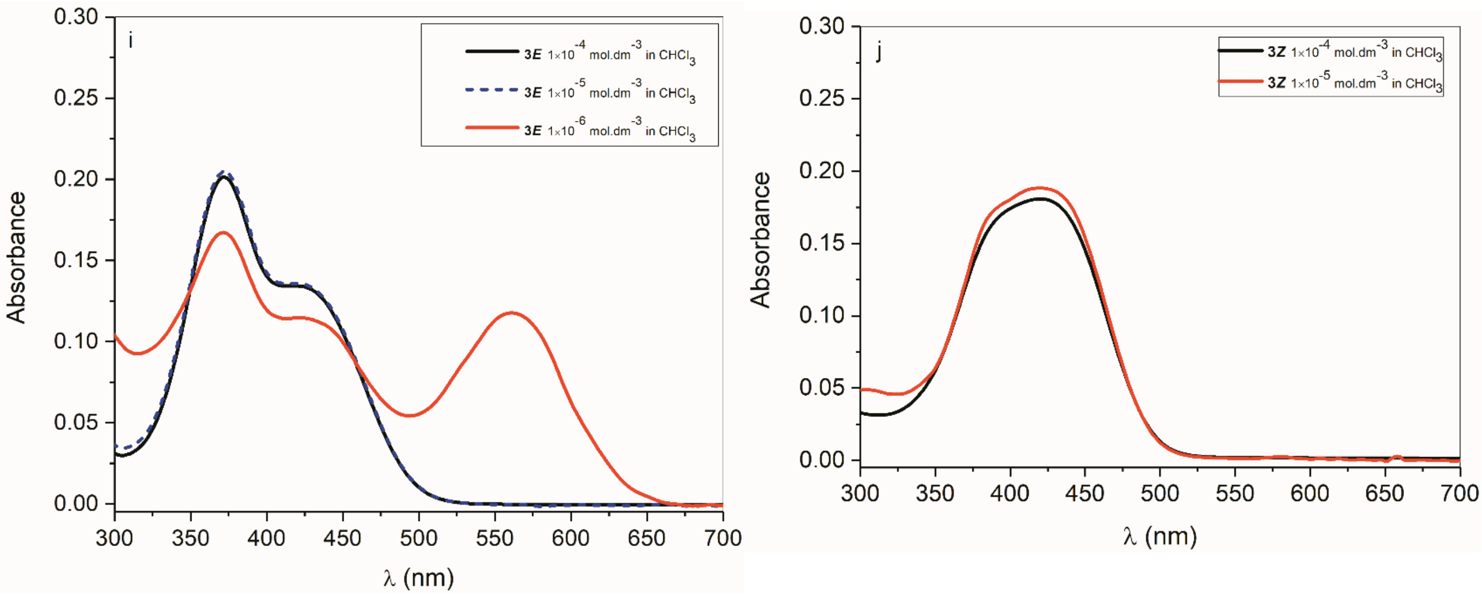

Figure S1. Concentration effect on UV-Vis spectra of *E*- and *Z*-isomers of hydrazones **1 *Z***, **2 *E***, **2 *Z***, **3 *E***, **3 *Z***, in CH_3CN and $CHCl_3$.

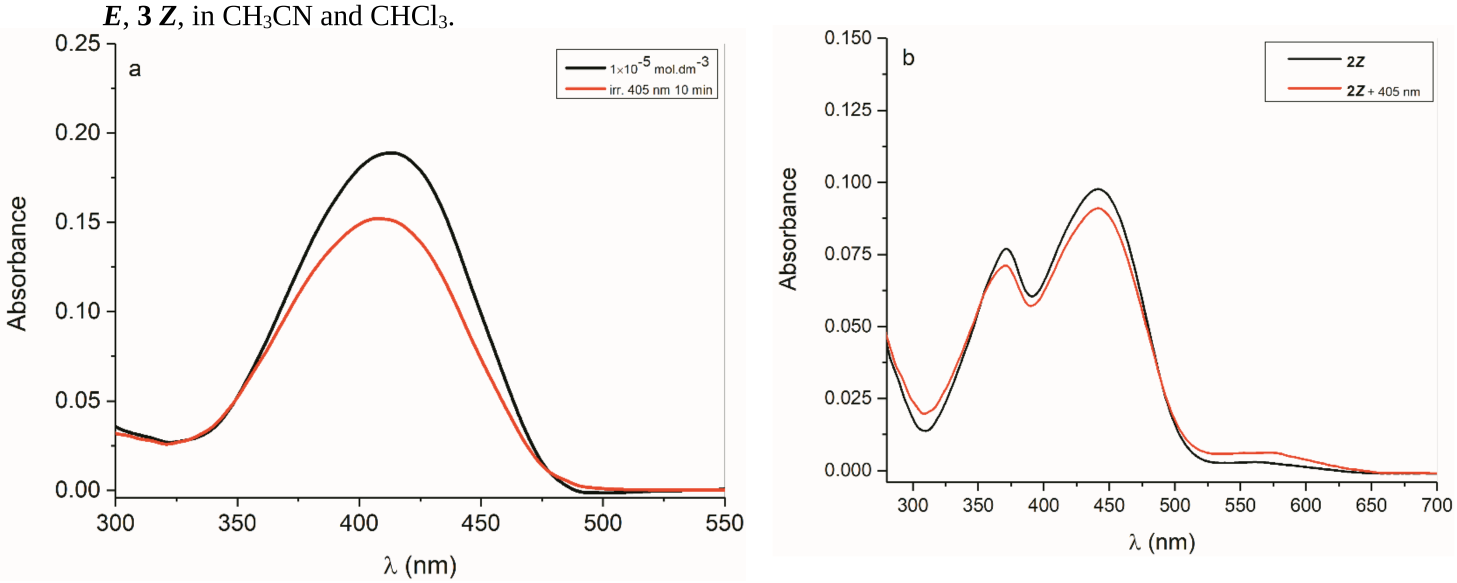

Figure S2. Change in UV-Vis spectra of hydrazones after irradiation with 405 nm light a) hydrazone **1** and b) hydrazone **2 *Z*** in DMF.

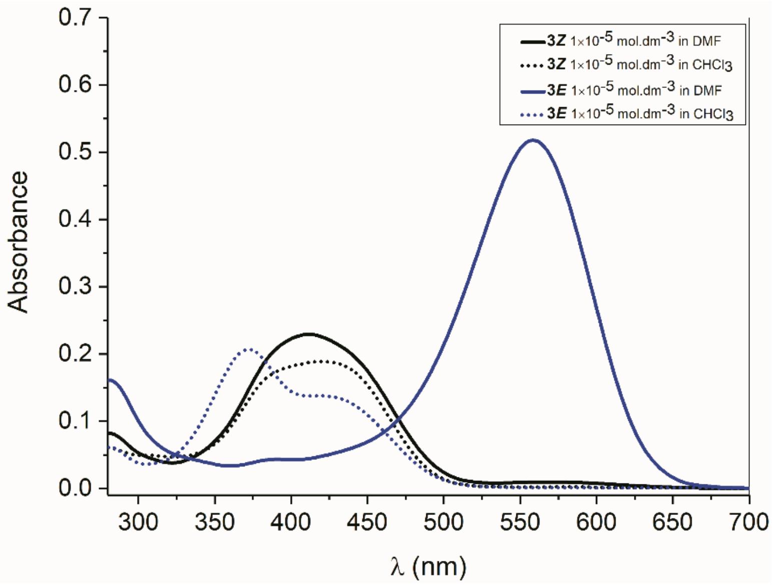

Figure S3. Effect of DMF and $CHCl_3$ on hydrazo=azo equilibrium of *E*- and *Z*-isomers of hydrazone **3** (1×10^{-5} $mol.dm^{-3}$).

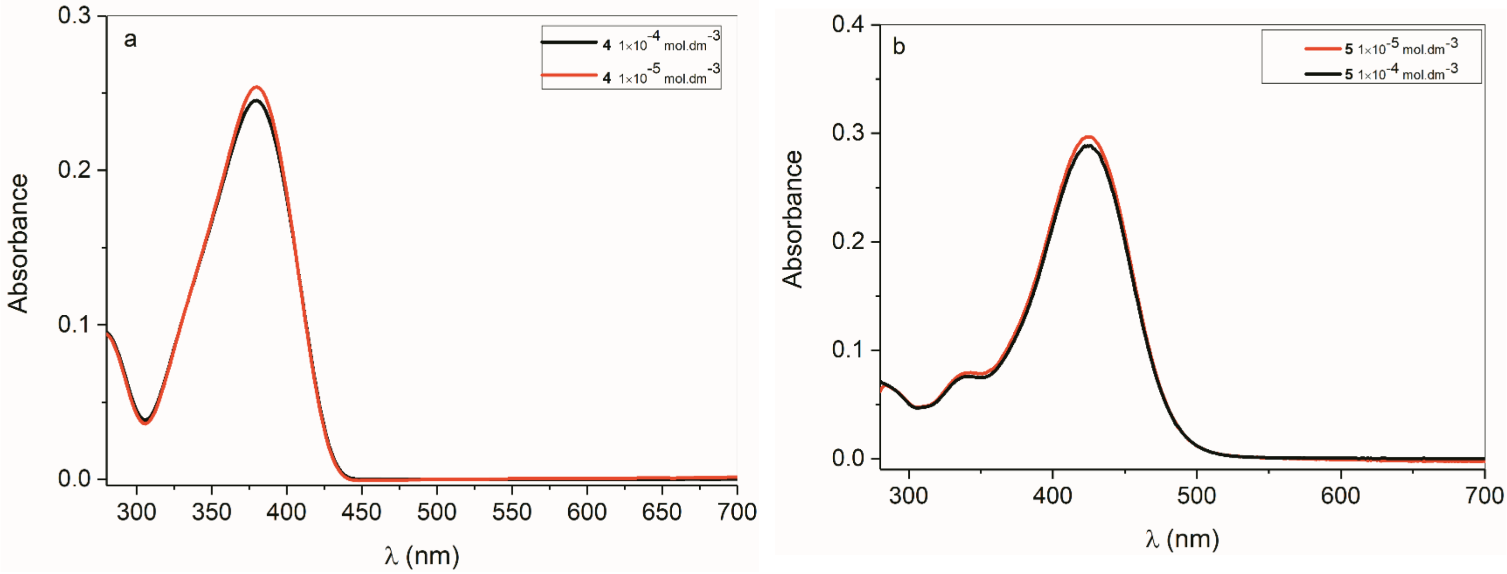

Figure S4. Concentration effect of compounds **4** and **5** on UV-Vis spectrum in DMF.

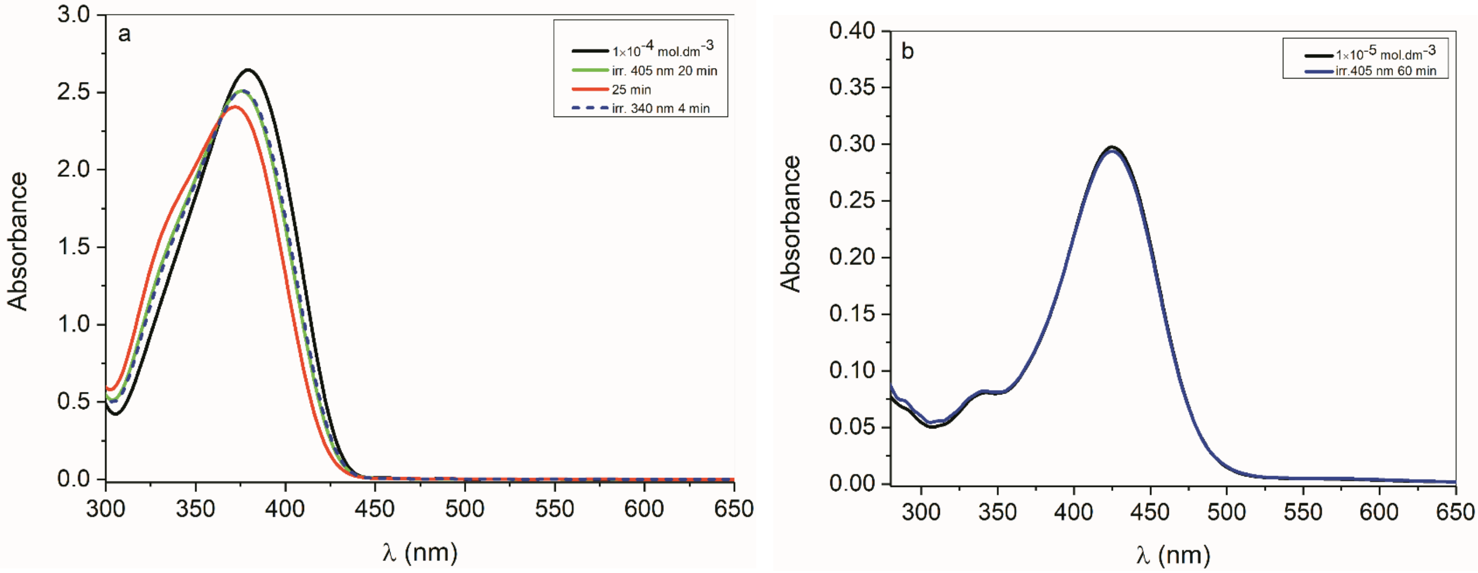

Figure S5. Photoisomerization of a) compound **4** b) compound **5** in DMF (1×10^{-5} mol.dm^{-3}).

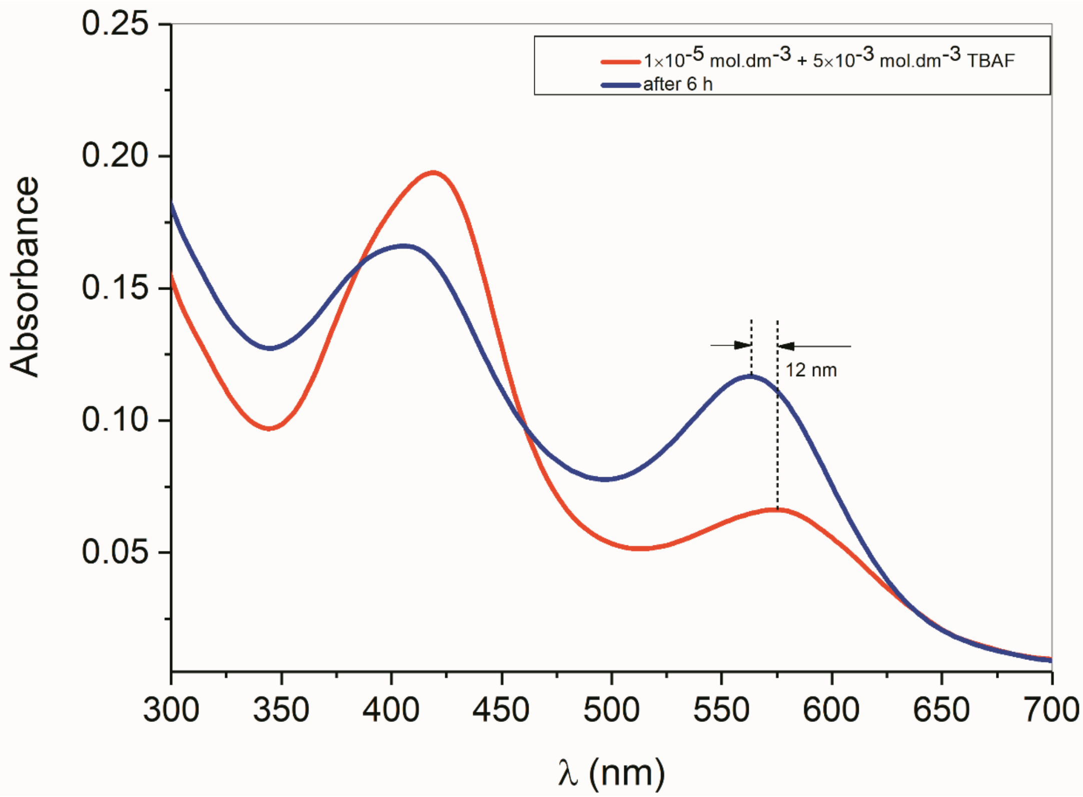

Figure S6. UV-Vis spectra change of hydrazone **3** (1×10^{-5} mol.dm^{-3}) depending on time at 25 °C in $CHCl_3$.

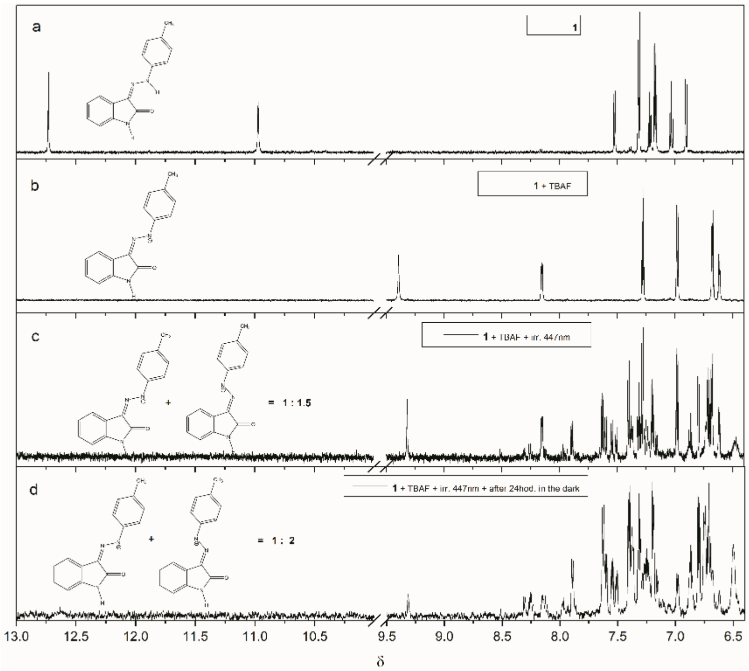

Figure S7. ^{1}H NMR spectrum of hydrazone **1** a) hydrazone **1** (1×10^{-3} mol.dm^{-3}); b) mixture a) + TBAF (5×10^{-5} mol.dm^{-4}); c) mixture b) after 20 h in the dark; d) mixture c) after irradiation with 405 nm light; e) mixture d) after irradiation with 445 nm light.

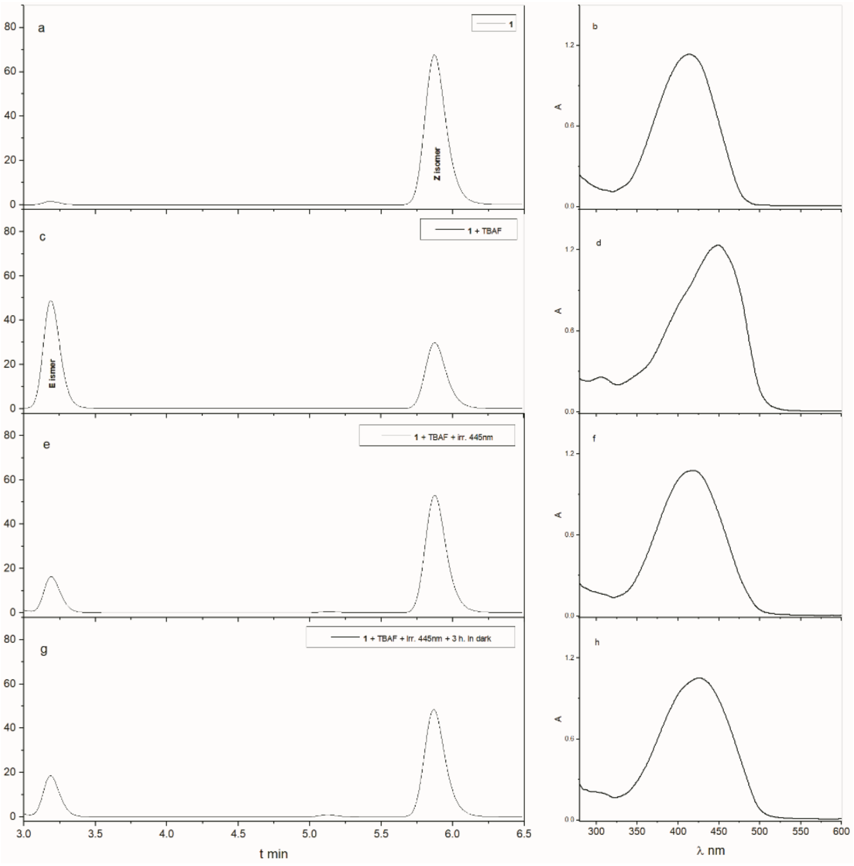

Figure S8. HPLC chromatogram and UV-Vis spectrum in DMF: a) and b) hydrazone **1** (5×10^{-5} mol.dm^{-3}); c) and d) hydrazone **1** + TBAF (3×10^{-4} mol.dm^{-3}); e) and f) mixture c) after irradiation with 470 nm light; g) and h) mixture e) after 16 h in the dark 25 °C.

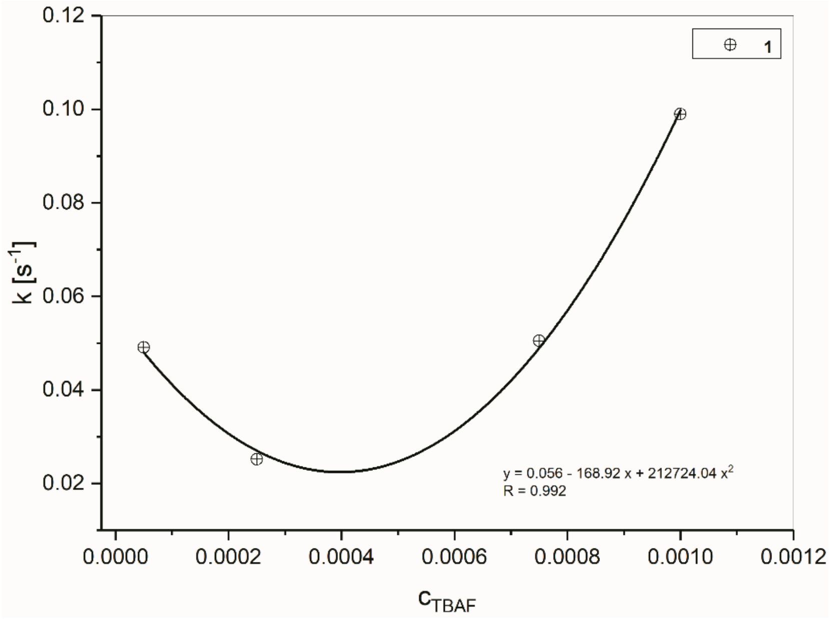

Figure S9. The rate constant k [s^{-1}] dependence of the process Z_{anion}=E_{anion} isomerization of hydrazone **1** anion (1×10^{-5} mol.dm^{-3}) on the concentration of TBAF in DMF at 25 °C.

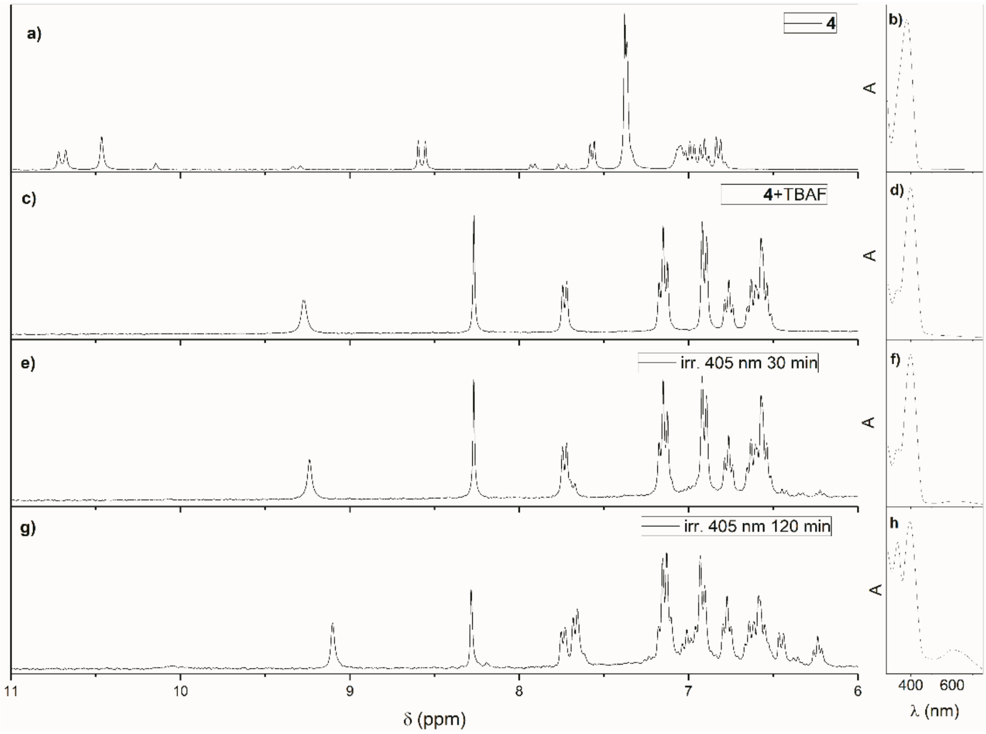

Figure S10. Photochemistry of compound **4** (1×10^{-3} mol.dm^{-3}) + TBAF (1×10^{-2} mol.dm^{-3}) monitored by ^{1}H NMR and UV-Vis spectra in DMSO.

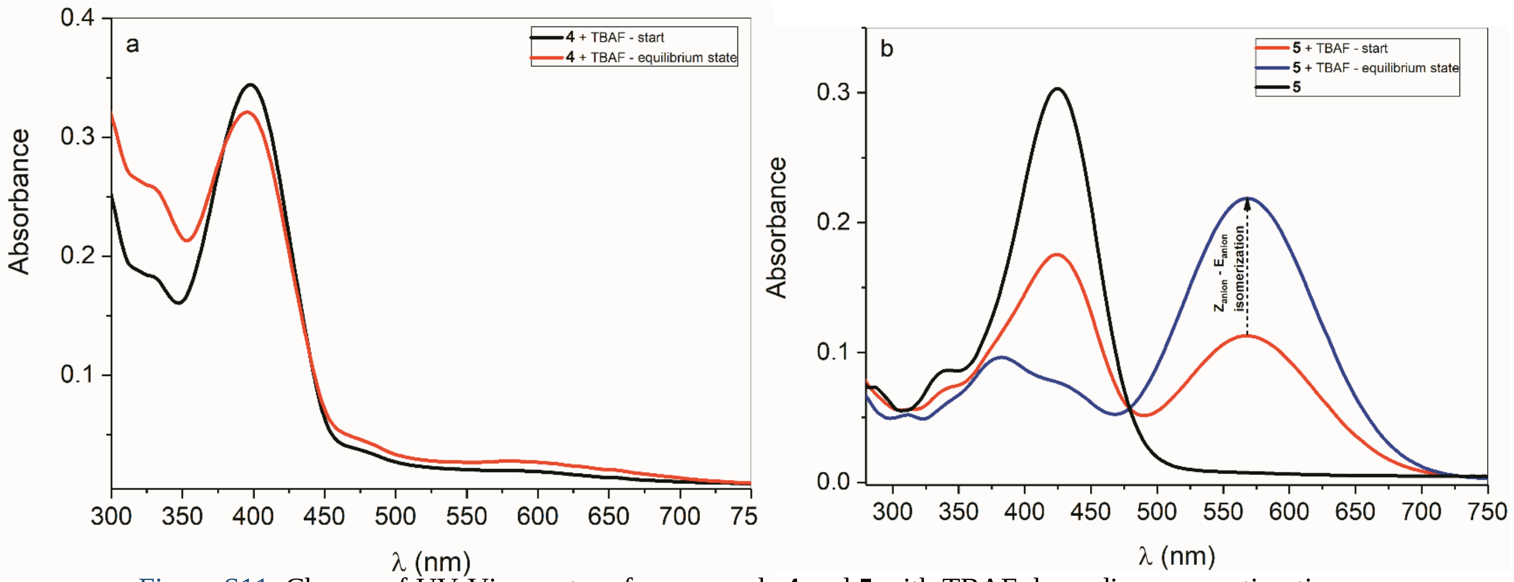

Figure S11. Change of UV-Vis spectra of compounds **4** and **5** with TBAF depending on reaction time 25 °C in DMF a) compound **4** (1×10^{-5} mol.dm^{-3}) + TBAF (1×10^{-2} mol.dm^{-3}); b) **5** (1×10^{-5} mol.dm^{-3}) + TBAF (1×10^{-4} mol.dm^{-3}).

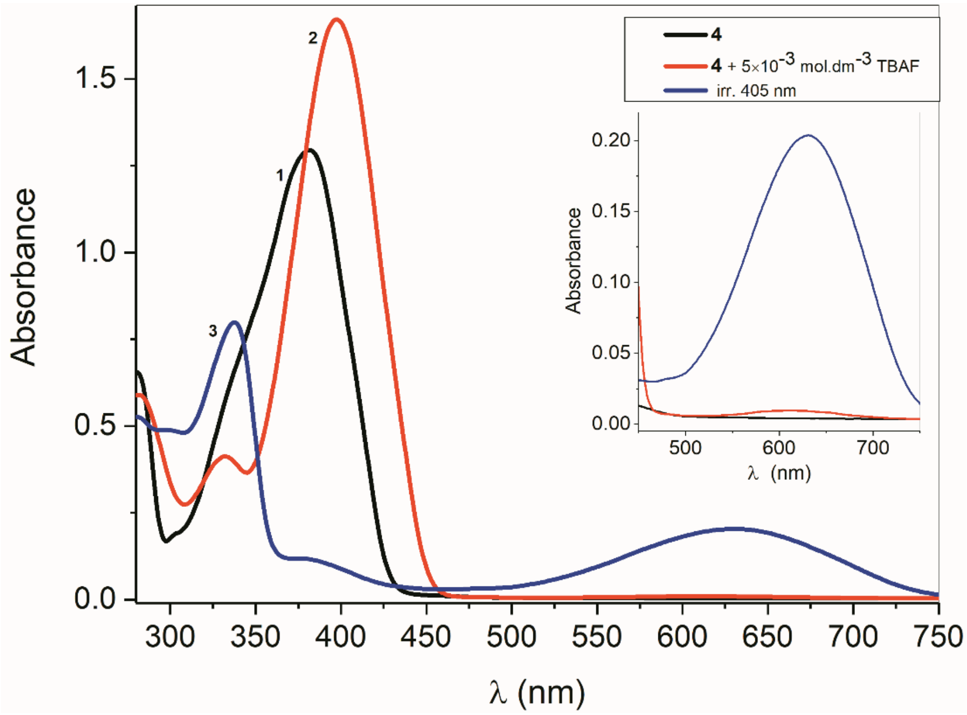

Figure S12. UV-Vis of compound **4** in DMF: 1– (**4** (5×10^{-5} mol.dm^{-3})); 2– (**4** + TBAF (5×10^{-3} mol.dm^{-3})); 3 – (**4** + TBAF (5×10^{-3} mol.dm^{-3}) after irradiation with 405 nm light).

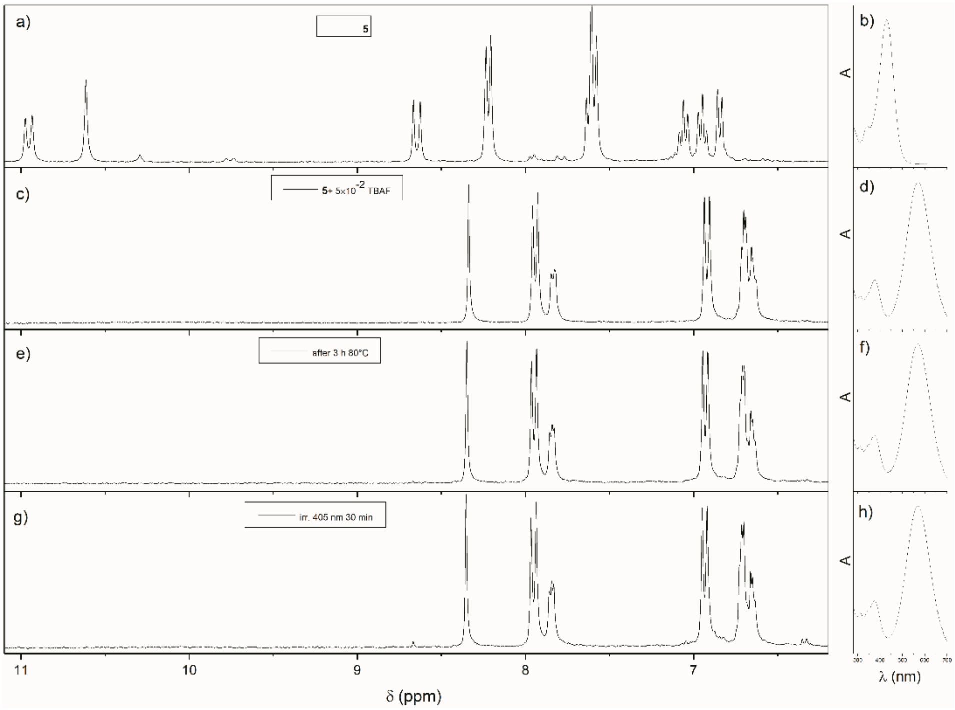

Figure S13. Effect of temperature and irradiation (405 nm) on compound **5** (5×10^{-3} mol.dm^{-3}) in the TBAF presence (5×10^{-2} mol.dm^{-3}) monitored by ^{1}H NMR and UV-Vis: a), b) compound **5**; c), d) **5** + TBAF; e), f) mixture c) after 3 h at 80 °C; mixture e) after irradiation with 405 nm light.

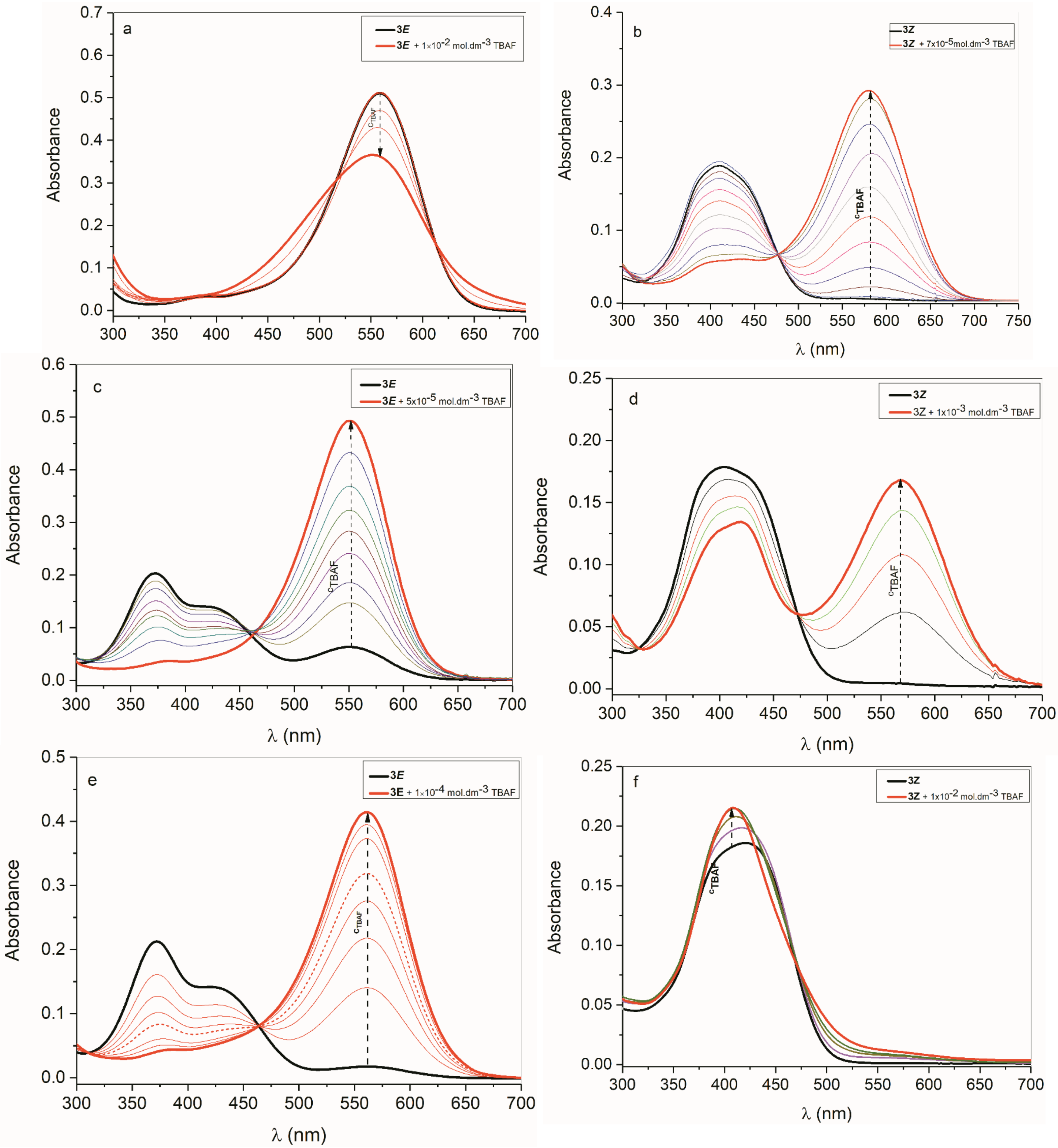
a
3E
3E + 1×10-2 mol.dm-3 TBAF
cTBAF
Absorbance
b
3Z
3Z + 7x10-5mol.dm-3 TBAF
cTBAF
Absorbance
λ (nm)
c
3E
3E + 5x10-5 mol.dm-3 TBAF
cTBAF
Absorbance
λ (nm)
d
3Z
3Z + 1x10-3 mol.dm-3 TBAF
cTBAF
Absorbance
λ (nm)
e
3E
3E + 1×10-4 mol.dm-3 TBAF
cTBAF
Absorbance
λ (nm)
f
3Z
3Z + 1x10-2 mol.dm-3 TBAF
cTBAF
Absorbance
λ (nm)

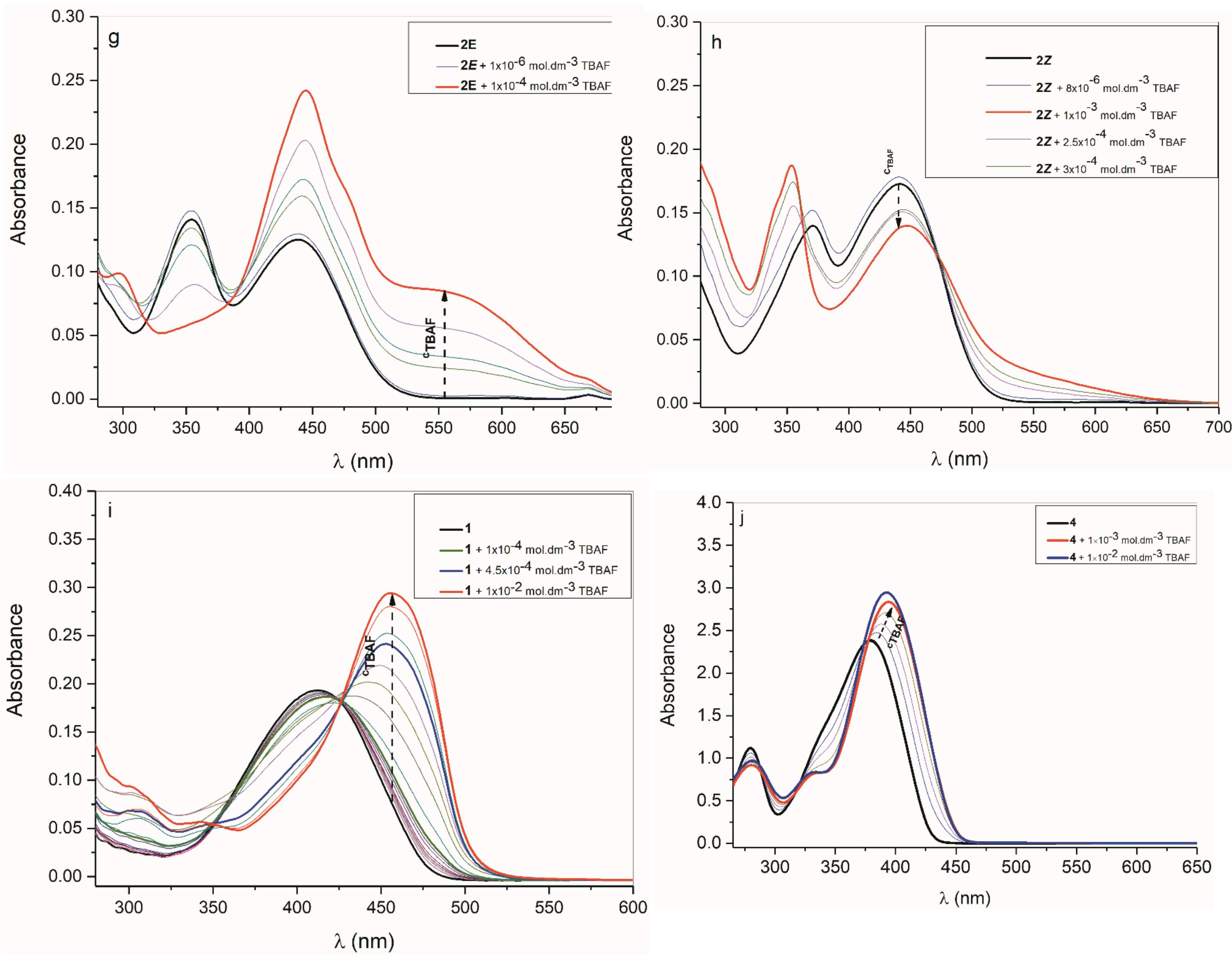

Figure S14. Titration of *E*- and *Z*-isomers of hydrazone: a) **3*E*** in DMF; b) **3*Z*** in DMF; c) **3*E*** in CH_3CN; d) **3*Z*** in CH_3CN; e) **3*E*** in $CHCl_3$; f) **3*Z*** in $CHCl_3$; g) **2*E*** in DMF; h) **2*Z*** in DMF; i) **1** in DMF; j) **4** in DMF.
